# Detection and characterisation of ligand-induced conformational changes in acetylcholine binding proteins using biosensors and X-ray crystallography

**DOI:** 10.1039/d5cb00041f

**Published:** 2025-08-13

**Authors:** Edward A. FitzGerald, Daniela Cederfelt, Daria Kovryzhenko, Pierre Boronat, Bjarte Aarmo Lund, Doreen Dobritzsch, Sven Hennig, Pablo Porragas Paseiro, Iwan J. P. de Esch, U. Helena Danielson

**Affiliations:** a Department of Chemistry – BMC, Uppsala University Sweden helena.danielson@kemi.uu.se; b Beactica Therapeutics AB Virdings allé 2 Uppsala Sweden; c Division of Medicinal Chemistry, Amsterdam Institute of Molecular and Life Sciences (AIMMS), Vrije Universiteit Amsterdam De Boelelaan 1108 1081 HZ Amsterdam The Netherlands; d Department of Chemistry, UiT The Arctic University of Norway Tromsø Norway; e Department of Chemistry and Pharmaceutical Sciences, Vrije Universiteit Amsterdam De Boelelaan 1108 1081 HZ Amsterdam The Netherlands; f Amsterdam Institute of Molecular and Life Sciences, Vrije Universiteit Amsterdam De Boelelaan 1108 1081 HZ Amsterdam The Netherlands; g Dynamic Biosensors GmbH Perchtinger Str. 8/10 81379 München Germany; h Science for Life Laboratory, Uppsala University Uppsala Sweden

## Abstract

Analysis of ligand-induced structural changes in proteins is challenging due to the lack of experimental methods suited for detection and characterisation of both ligand binding and induced structural changes. We have explored biosensors with different detection principles to study interactions between ligands and acetylcholine binding proteins (AChBPs), soluble homologues of Cys-loop ligand gated ion channels (LGICs) that undergo similar structural changes as LGICs upon ligand binding. X-ray crystallography was used to identify binding sites and establish if the detected conformational changes involved small changes in loop C or major structural changes in the pentamer associated with ion channel opening. Experiments were initially focused on ligands exhibiting complex surface plasmon resonance (SPR) biosensor sensorgrams or detected by second harmonic generation (SHG) biosensor analysis. Surface acoustic wave (SAW) and SHG biosensors confirmed that complexities in SPR data were indeed due to ligand-induced conformational changes. Grating coupled interferometry (GCI) biosensor sensorgrams were less complex, despite similar detection principles. switchSENSE biosensor analysis revealed that ligands resulted in either a compaction or expansion of the protein structure. X-ray crystallography of the protein–ligand complexes was only successful for 7 out of 12 ligands, despite nM–μM affinities. Crystals were not obtained for the two compounds shown by SHG analysis to induce large structural changes, while electron densities were not seen in the structures for some ligands. The work presented herein shows that several biosensor technologies have a unique capability to detect and discriminate binding and ligand induced conformational changes in proteins, also when interactions are rapid, weak and structural changes are small. However, they are complementary and provide different information.

## Abstract 2

Analysis of ligand-induced structural changes in proteins is challenging due to the lack of experimental methods suited for detection and characterisation of both ligand binding and induced structural changes.

## Graphical abstract

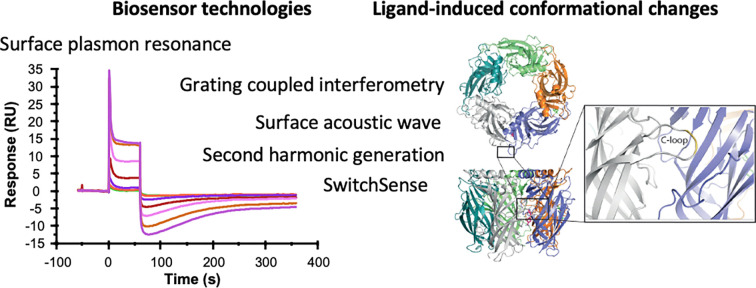

## Introduction

The Cys-loop ligand gated ion channel (LGIC) superfamily of pentameric membrane-bound receptors is primarily involved in neurotransmission in the central and peripheral nervous systems, but other functions are also known.^1^ LGICs are activated by ligands binding at subunit interfaces of their extracellular domains, thereby inducing conformational changes that affect the structure of a central pore and its ability to transport ions through the membrane.^2^ The Cys-loop is a functional motif formed by a disulfide bond between two cysteine residues near the N-terminus. Conformational changes in the Cys-loop are critical for the function of the ion channel, since its movement away from the pore axis causes tilting of the helix bundle, which results in channel opening.^3^ Several inherited neurological disorders such as epilepsy are associated with mutations in Cys-loop receptors and many LGICs are of importance as targets for anaesthetics, benzodiazepines and other drugs. Such compounds can have direct functional effects by binding directly to the orthosteric site of the LGIC, *via* essentially native modes-of-action, or indirectly *via* binding to allosteric sites and involving novel modes-of-action.

Identifying new regulators of LGICs with particular modes-of-action and novel therapeutic effects is challenging due to the lack of biochemical and biophysical methods for this class of proteins. Although cell-based assays provide important functional effects and can differentiate agonists from antagonists, they do not provide the mechanistic or structural details for rational design of functional lead compounds. Methods that can locate their binding sites and characterise their binding modes and induced conformational changes need to be combined with methods that can quantify the kinetics of the interactions and subsequent conformational changes. For therapeutic discovery, methods should be suitable for screening and identification of ligands based on their potential to be developed into efficient and safe agonists or antagonists.

**Fig. 1:**
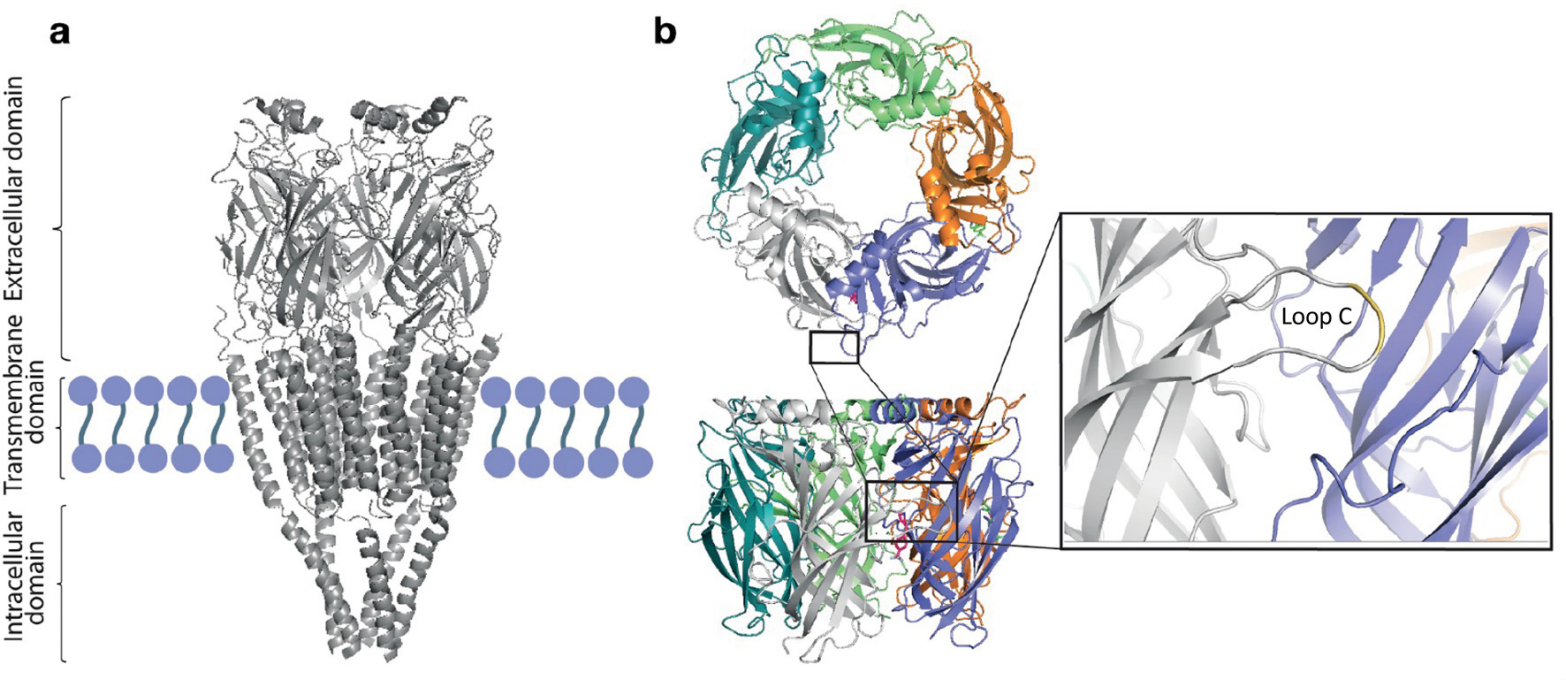
Structure of (a) the nicotinic acetylcholine receptor, a pentameric LGIC with extracellular, transmembrane and intracellular domains (PDB: 2BG9), (b) *Ls*-AChBP, corresponding to the extracellular domain of a pentameric LGIC. The highly conserved loop C is highlighted (inset) (PDB: 1UW6).

The orthosteric ligand binding site of nAChR binds agonists, partial agonists and antagonists, located between the subunits (Fig. 1).^4,5^ Although they bind to the same site, agonists and antagonists result in different structural changes and functional effects. Crystal and electron microscopy structures have shown that agonists induce a clockwise rotation of the inner sheets in the N-terminal domains of two α subunits, followed by an inward movement of loop C (“loop C capping”) in the extracellular domain, which tightens the binding pocket.^2,6–8^ Conversely, antagonists push loop C in the opposite direction, thus opening the pocket. Molecular dynamics simulations have provided important insights into LGIC structure and function.^9^

A complicating feature for analysis of this regulatory mechanism is that it is a two-step process, with complex formation followed by a conformational change (reaction (R1), P: protein, L: ligand).

R1 P + L ⇄ PL ⇄ PL*

There is no correlation between the kinetics and affinity of the first step and the kinetics or magnitude of the conformational rearrangement in the second step. Since the kinetics of both steps, as well as the functional effect of the second step, *i.e.* channel opening or not in nAChRs, are of importance, a full characterisation of the mechanism requires a time-resolved biophysical method capable of distinguishing P, PL and PL*.

To enable a biophysical approach for the discovery of ligands targeting LGICs, we have previously used native and engineered acetylcholine binding proteins (AChBPs). These are suitable as model systems for studying ligand-binding and structural rearrangements in LGICs.^10–13^ AChBPs are homopentameric, water-soluble homologs of the ligand-binding domain of LGICs (Fig. 1(b)) and well-established structural surrogates for nicotinic acetylcholine receptors (nAChR).^14–16^ Herein, we have used AChBP from *Lymnea stagnalis* (*Ls*-AChBP) and *Aplysia californica* (*Ac*-AChBP). They have a sequence identity to each other of 33%, and of 25% and 26% to the nAChR, respectively. Importantly, AChBPs can bind nAChR-specific ligands and induce similar conformational changes as in the membrane bound receptors.^4,13^

Surface plasmon resonance (SPR) based biosensor assays have allowed the detection and characterization of compounds interacting with AChBPs,^10,11,17^ engineered chimeric binding proteins (5HTBP: *Ac*-AChBP and serotonin receptor (5-HT_3_ R),^18^ α7-AChBP: *Ac*-AChBP and α7 nicotinic acetylcholine receptor (nAChR))^12^ and full length β3 GABA_A_ receptors.^19^ The time-resolved sensorgrams for these interactions are complex and not well described by a simple Langmuir model for a reversible binary (1 : 1) interaction in one step. The complexity observed in the data has been attributed to changes in the refractive index of the surface due to changes in the hydrodynamic radius of the immobilized protein.^20,21^ Although the data reveal that ligands induce conformational changes upon binding, it is not possible to characterise the mechanism, kinetics or structural features from this type of data alone.^10,17^ To confirm that the complexities were due to conformational changes and not experimental artifacts, we have also used a Second Harmonic Generation (SHG) based biosensor to study ligand interactions with AChBPs.^22^ The nAChR agonists varenicline, epibatidine, lobeline and the antagonist tubocurarine, all gave SHG signals, which differed for specific ligand classes.^22^ The SHG biosensor analysis was very sensitive and importantly, when used for screening of a fragment library, hits were detected on the basis of induced conformational changes, despite weak affinities.

Herein, we explore additional biosensor technologies for studies of ligand induced conformational changes in AChBPs. The range of biosensor types used differ in their detection principles and the experimental design that can be used. This affects their capability to provide qualitative and/or quantitative information about the ligand–protein interaction, as well as their ability to detect and provide information about ligand-induced structural changes. A major challenge in these experiments was the low molecular weight and weak affinities of the ligands, and the small structural changes induced upon binding, requiring very high sensitivities.

## Results

**Fig. 2:**
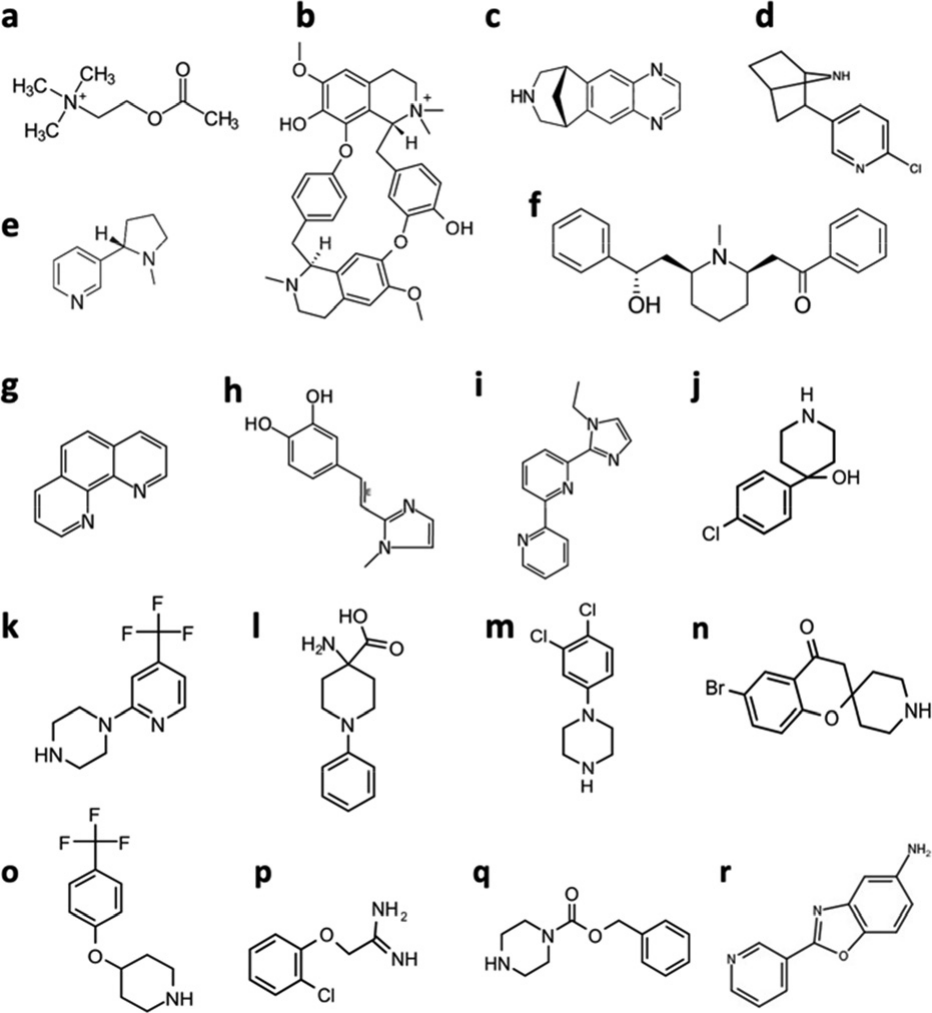
Chemical structures of utilized compounds: (a) acetylcholine, (b) tubocurarine, (c) varenicline, (d) epibatidine, (e) nicotine, (f) lobeline, (g) VUF6105, (h) VUF22430, (i) VUF24234, (j) FL3044, (k) FL1613, (l) FL1909, (m) FL1971^22^ (n) FL1856,^22,23^ (o) FL1888,^22,23^ (p) FL8561, (q) FL8454 and (r) FL1961. VUF-series compounds are from the Vrije university fragment library.^24^ FL-series compounds are from the Uppsala university/SciLifeLab fragment library.^23^

Compounds previously identified in SPR- or SHG-based screening campaigns^22,23^ and three new compounds found to give complex SPR sensorgrams, were selected for analysis of their interactions with *Ls*-AChBP and *Ac*-AChBP using different types of biosensors and X-ray crystallography (see the Discussion section for a brief description and comparison of the data from the different biosensors). In addition, a set of natural nAChR agonists/antagonists was used as tool ligands: acetylcholine, nicotine, epibatidine, tubocurarine, varenicline, and lobeline. Their interactions with both forms of AChBP have previously been characterized using biosensors, so they were considered to be suitable as references and to confirm that AChBP was appropriately folded and functional over time in the experiments.^10,12,22,23,25^ All studied compounds are shown in Fig. 2.

### Surface plasmon resonance (SPR) biosensor analysis

**Fig. 3:**
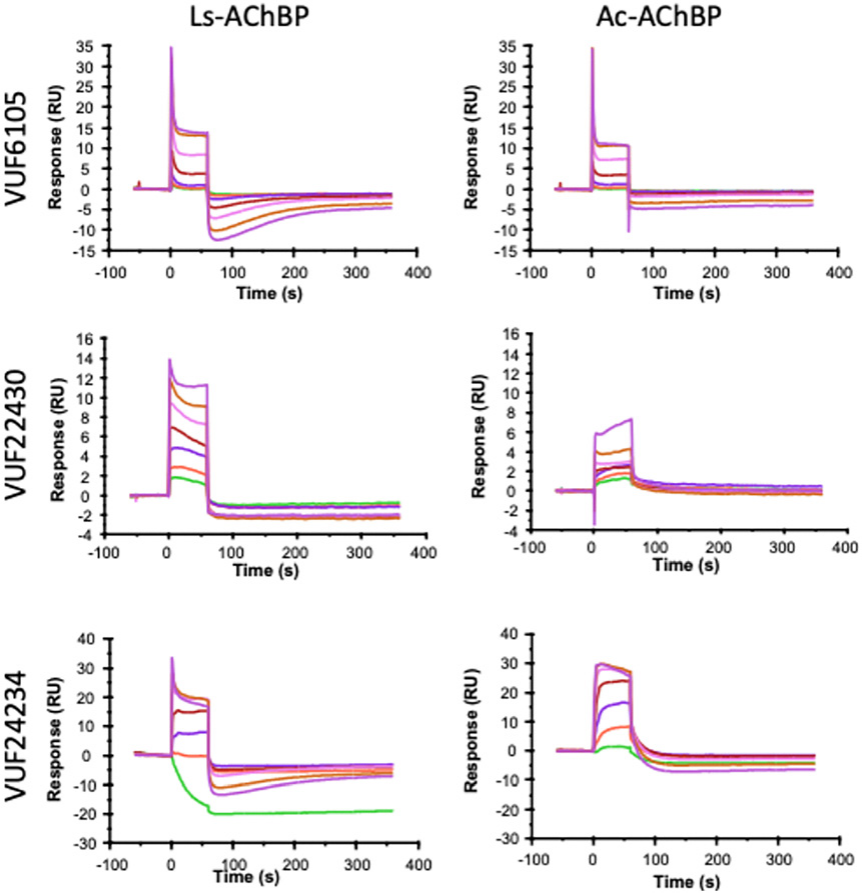
SPR biosensor sensorgrams for VUF6105, VUF22430 and VUF24234 interacting with immobilised *Ls*-AChBP and *Ac*-AChBP. Ligands were injected in a serial two-fold dilution series, starting at 100 μM. Sensorgrams were double referenced.

The starting point of the project was complex SPR biosensor data obtained for three previously uncharacterised compounds: VUF6105, VUF22430 and VUF24234 (Fig. 2(g)–(i)), and immobilised *Ls*-AChBP and *Ac*-AChBP. The sensorgrams had concentration dependent distortions in both the association and dissociation phases, with a negative slope at steady state and signals below baseline once the ligand injection was stopped (Fig. 3).

The three compounds all induced secondary effects upon binding, shown as a deviation from a single exponential signal in the association and dissociation phases, and often negative signals before returning to baseline. The data are double referenced and the distortions are consequently not due to binding to the reference surfaces, which in all cases was negligible. The sensorgrams cannot be used for estimation of kinetic parameters or affinities since it is not possible to define an interaction model suitable for global non-linear regression analysis of sets of sensorgrams or steady state *vs.* concentration datasets. Still, it can be concluded from the shape of the sensorgrams that both the association and dissociation rate constants are very fast, and that secondary effects occur on a much slower timescale. The secondary effects remained after the ligand had dissociated indicating that they represent characteristics of the ligand–protein complex and not the ligand alone. In addition, the effects were different for the three compounds and the two forms of AChBP, as expected for specific effects related to the different structures of both the ligands and the proteins.

As a complement, we returned to a set of fragments identified in an SPR-based fragment screening campaign against *Ls*-AcCBP (Fig. S1).^23^ We selected two fragments (FL1856 and FL1888) that had slower dissociation rates than characteristic for fragments (Fig. S1a and b). Although these compounds fulfil basic hit criteria, they are typically omitted from further evaluation as they indicate that the compounds are “sticky” or interact with the target with a complexity that makes fragment evolution challenging. However, the observed binding complexity revealed that these compounds potentially induced functional effects *via* structural rearrangements in the receptor. We were consequently interested in confirming that the distortions were indeed due to ligand-induced conformational changes. For comparison, we also included a set of fragment hits from the same screen, but that did not show secondary effects in the SPR sensorgrams (Fig. S1c–f), while lobeline resulted in complex sensorgrams for *Ac*-AChBP, but not *Ls*-AChBP (Fig. S1g).^23^

### Grating coupled interferometry (GCI) biosensor analysis

**Fig. 4:**
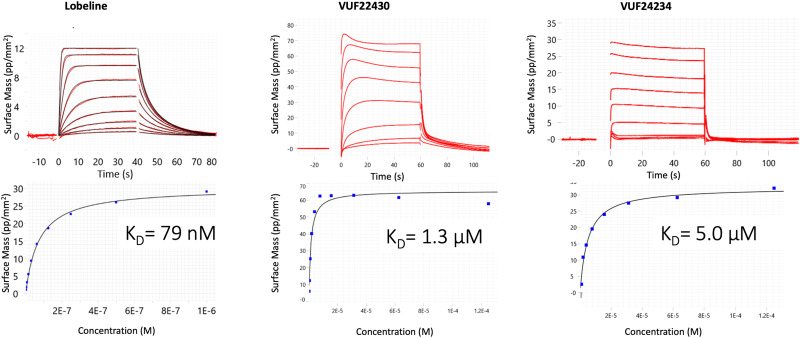
GCI data for lobeline, VUF22430 and VUF24234 interactions with *Ls*-AChBP. Sensorgrams for concentration series and the corresponding steady state signals *vs.* concentration plots with estimated *K*_D_-values based on a 1 : 1 model. Note the lower concentration range used for the lobeline injections. Sensorgrams were double referenced.

For comparison, a Grating Coupled Interferometry (GCI)-based biosensor was used to analyse interactions between VUF22430 and VUF24234 with *Ls*-AChBP (Fig. 4), using an identical experimental design and lobeline as a reference. The GCI sensorgrams were not as complex as those for the SPR analyses and a visual inspection of the sensorgrams revealed that both VUF22430 and VUF24234 had lower affinities and much faster interactions than lobeline, but exhibited secondary effects at higher concentrations. Although the data for lobeline did not appear to be complex, the *K*_D_-values from a global fit and a steady state analysis of the sensorgrams were slightly different (Fig. S2), indicating that also the lobeline interaction was more complex. Since no suitable interaction model is available, *K*_D_-values were estimated by steady state analysis using a 1 : 1 model, although it disregards the observed secondary effects. The estimated affinities for VUF22430 and VUF24234 were much lower (*K*_D_ = 1.3 and 5 μM) than for lobeline, reflecting faster dissociation rates.

### Surface acoustic wave (SAW) biosensor analysis

Surface Acoustic Wave (SAW) biosensor technology was used to confirm that distortions observed in SPR biosensor data (Fig. 3) for interactions between compounds VUF6105, VUF22430, and VUF24234, and AChBP indeed result from ligand-induced conformational changes. These experiments were preliminary and could not be optimised due to limited access to the instrument. Nevertheless, the analysis using acetylcholine, VUF6105, VUF22430, and VUF24234*vs.* AChBP showed proof-of-principle for this technology.

**Fig. 5:**
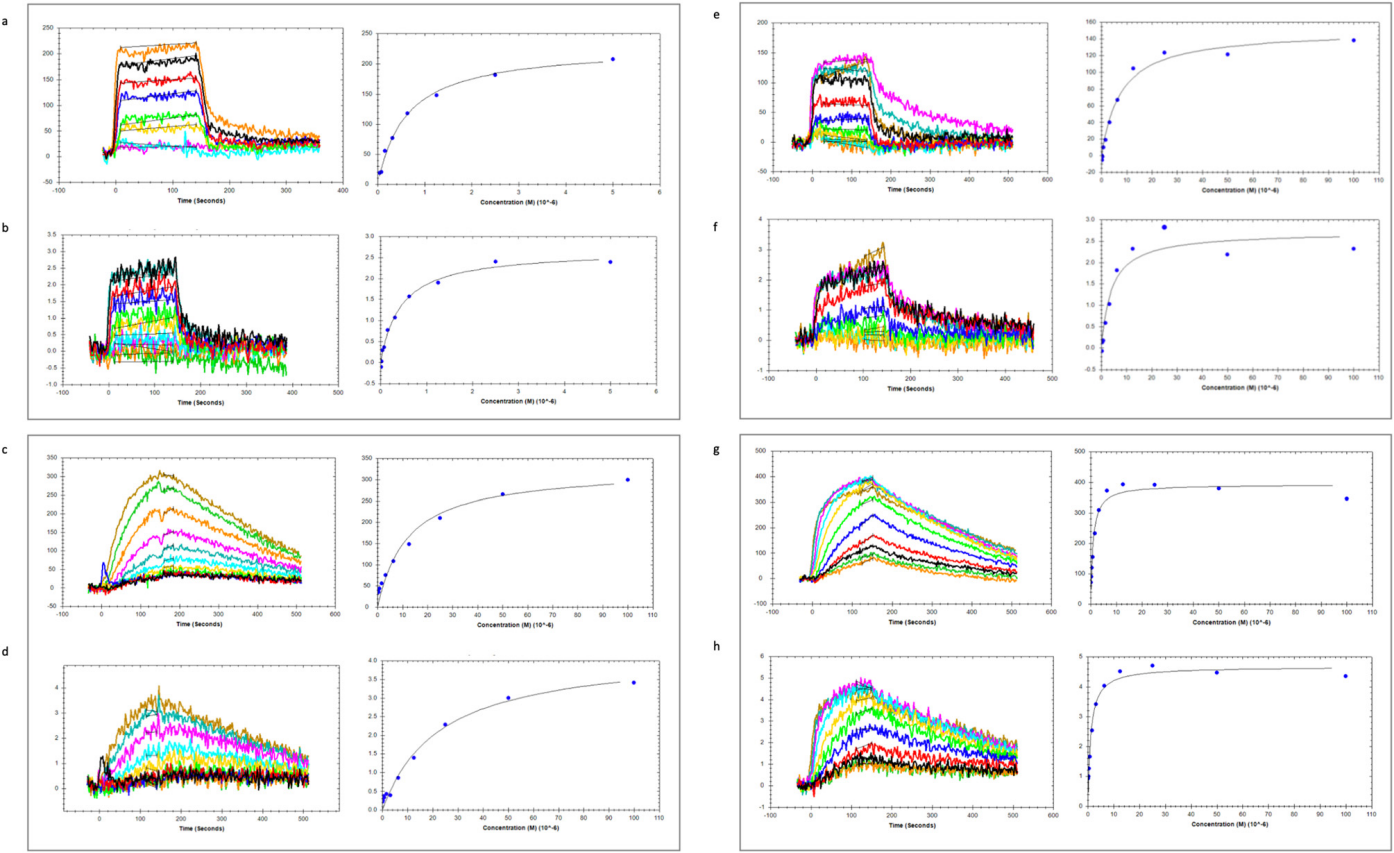
SAW biosensor analysis of ligand interactions with *Ls*-AChBP. Phase signals representing the binding event are in panels a, c, e and g, while amplitude signals representing the conformational change are in panels b, d, f and h. (a) and (b) 0–5 μM acetylcholine, (c) and (d) 0–100 μM VUF6105, (e) and (f) 0–100 μM VUF22430 and (g) and (h) 0–100 μM VUF24234. See Fig. S3 for the data for these compounds and nicotine with both *Ls*-AChBP and *Ac*-AChBP, the data were used for estimation of *K*_D_ and apparent *K*_D_ values (Table 1).

Both types of responses expected for a ligand that binds (phase signal) and induces conformational changes (amplitude signal) in an immobilised protein were observed (Fig. 5). Nicotine and the three VUF compounds all showed both detectable phase and amplitude signals for both forms of AChBP, while acetylcholine and VUF22430 only gave detectable signals for *Ls*-AChBP (Fig. S3) (see Fig. S3 for data for these compounds and nicotine*vs.* both *Ls*-AChBP and *Ac*-AChBP)

The signals were typically very noisy, and in some cases very weak, but a clear time and concentration dependence was observed. Global, non-linear regression analysis with a relevant interaction model could not be carried out due to the poor quality of the data. Although steady state was not reached for all interactions, it was possible to fit a 1 : 1 Langmuir interaction model to both the phase and amplitude data (Fig. 5 and Table 1).

**Table 1:** Equilibrium constants for ligand interactions with *Ls* and *Ac*-AChBP based on SAW data (from Fig. 5 and Fig. S3). *K*_D_-values were estimated from the signals at steady state *vs.* concentration from phase data, and apparent *K*_D_-values from amplitude data

Compound	Protein	Phase *K*_D_ (μM)	Amplitude apparent *K*_D_ (μM)
Acetylcholine	*Ls*-AChBP	0.61	0.45
	*Ac*-AChBP	—	—
Nicotine	*Ls*-AChBP	0.035	0.033
	*Ac*-AChBP	0.79	1.0
VUF6105	*Ls*-AChBP	12	23
	*Ac*-AChBP	39	11
VUF22430	*Ls*-AChBP	7.6	4.1
	*Ac*-AChBP	—	—
VUF24234	*Ls*-AChBP	0.89	1.1
	*Ac*-AChBP	1.2	3.3

The phase data were used for estimation of the *K*_D_ of the complex, *i.e.* step 1 in the reaction (reaction (R1)). An apparent *K*_D_ value was estimated from the amplitude data. Since it describes the equilibrium of the two states of the complex, *i.e.* step 2 in the reaction (reaction (R1)), this has a very different meaning, although the values appear to be correlated (Table 1).

### Second harmonic generation (SHG) biosensor analysis

**Fig. 6:**
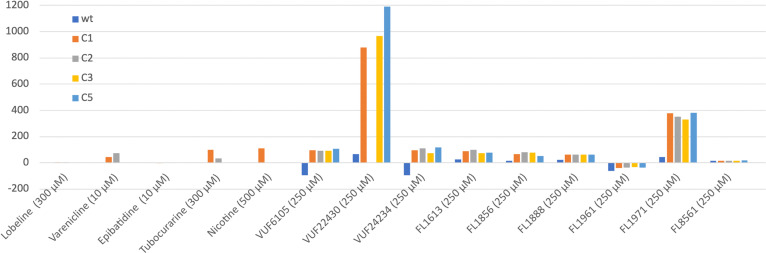
ΔSHG responses (% relative to a baseline response of blank injections) for ligands interacting with wild type *Ls*-AChBP labelled with SHG dye *via* K158, or engineered *Ls*-AChBP with specifically introduced cysteine residues: K98C (C1), K138C (C2), K178C (C3), S306C (C5), from left to right (see Fig. S4 for concentration response curves (CRC) and time course data).

A second harmonic generation (SHG) biosensor was used for direct detection of changes in the structure of the protein, upon binding of the selected compounds. The analysis was done using wild-type *Ls*-AChBP, which has the SHG dye conjugated to a lysine residue in the ligand binding site, and engineered *Ls*-AChBP, to which the dye was conjugated to cysteine residues introduced at specific sites.^22^ The C1, C2 and C3 proteins are labelled in the core of the protein, while the C5 protein is labelled at the C-terminus, in the region that corresponds to the membrane interaction domain on full length LGICs. The known nAChR ligands varenicline, epibatidine, lobeline, tubocurarine and nicotine were used as references. Changes in the SHG signal (ΔSHG) are shown in Fig. 6. Note the logarithmic *y*-axis and the differences in concentrations for the different compounds!

The three VUF compounds resulted in significant ΔSHG responses with all labelling variants, with VUF22430 giving very high signals. Five out of the six FL-series fragments analysed gave clear responses. FL1971 gave much higher signals than all other fragments and FL1961 differed in that it only gave negative responses. Only FL8561 did not give any significant responses. The responses were consistently lower for the wt-labelled protein, compared to the C1, C2, C3 and C5 variants. FL1971 gave the strongest signals, followed by FL1971. For comparison, lobeline and epibatidine did not result in any signal against any variant at the concentrations used, while varenicline, tubocurarine and nicotine all gave signals with the C1-labelled protein. Varenicline and tubocurarine gave low signals also for the C2 protein.

### switchSENSE biosensor analysis

**Fig. 7:**
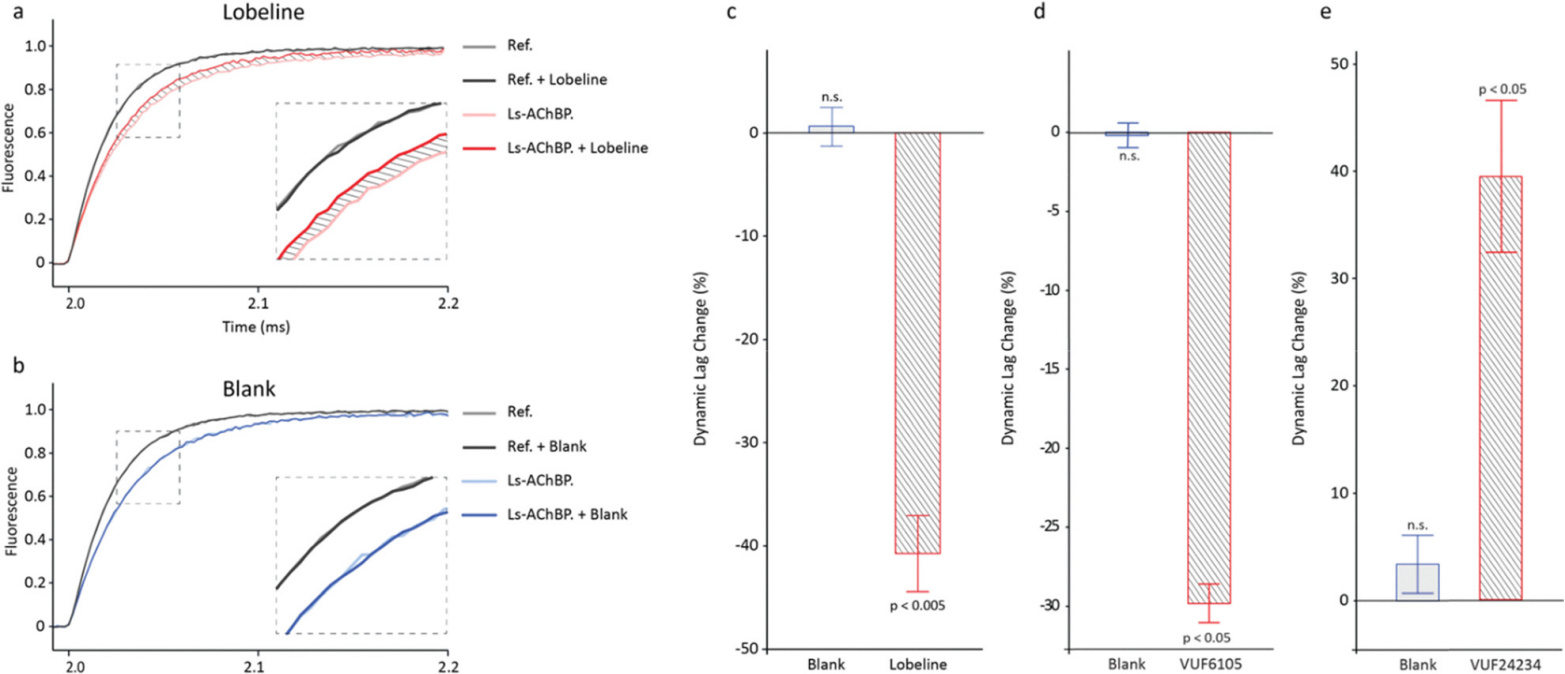
Upwards motion of DNA nanolevers without protein and with *Ls*-AChBP attached to the distal end (a) before and after lobeline injection or (b) before and after blank injection. (c) Dynamic lag change values of *Ls*-AChBP calculated from the upwards motion curves in a and b upon blank and lobeline injections. Error bars represent standard error of the mean of triplicate measurements. Statistical significance was calculated using a Welch's *t*-test with triplicate measurements. (d) DLS for VUF6105 and (e) DLS for VUF24234.

Finally, the potential of switchSENSE technology to detect small changes in the structure of *Ls*-AChBP upon ligand binding was explored using lobeline, VUF6105, VUF24230 and VUF24234. Lobeline was used for establishment of a reliable assay and as a benchmark compound. Binding of lobeline to *Ls*-AChBP resulted in a clear increase in nanolever motion speed towards the upright position, highlighted by the shaded area in Fig. 7(a). In contrast, a blank injection had no effect on the motion speed of the nanolever, yielding an insignificant Dynamic Lag Change (DLC) (Fig. 7(b)). Further analysis, including referencing to a DNA nanolever without tethered *Ls*-AChBP, yielded a DLC of approximately −40% (Fig. 7(c)). This indicates that lobeline induces a compaction of *Ls*-AChBP. Similarly, VUF6105 resulted in a significant compaction, while VUF24234 resulted in an expansion. No reliable data were obtained for VUF24230 since it was found to interact with the DNA nanolever itself (Fig. S5c).

As seen from the different conformational changes induced by each compound, this analysis revealed that the binding modes of VUF6105 and VUF24234 are different. Based on similar signals, it is possible that the binding mode for VUF6105 resembles the binding mode for that of lobeline.

### X-ray crystallographic analysis of conformation changes

**Fig. 8:**
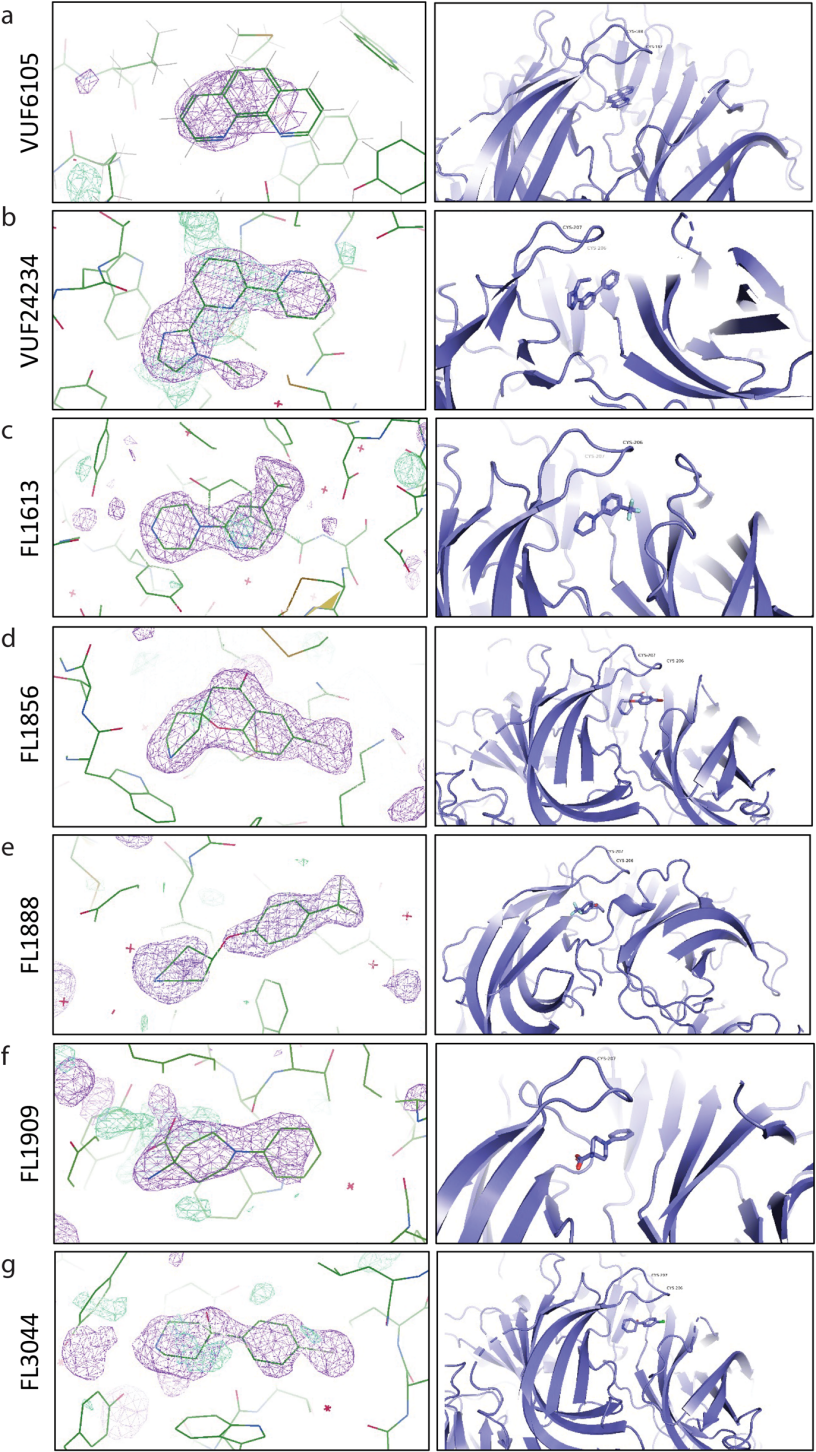
Structures of binding pockets at the subunit interface of *Ls*-AChBP in complex with (a) VUF6105 (9SG3.PDB), (b) VUF24234 (8P22.PDB), (c) FL1613 (8P1E.PDB), (d) FL1856,^22^ (e) FL1888,^22^ (f) FL1909 (8P1F.PDB), and (f) FL3044 (8P11.PDB). Simulated-annealing composite omit map (*m*F_o_–*D*F_c_) contoured at 1 RMSD (purple) used to model fragments into the density.

To investigate if the conformational changes detected by biosensor analyses could be pinpointed in the AChBP structure and the binding modes for different compounds be compared, co-crystallization of *Ls*-AChBP and the three VUF compounds as well as seven fragments was performed. (The structures of two of the fragments have been determined previously.^22^) Despite trying various strategies, no diffracting crystals were obtained for *Ls*-AChBP in complex with VUF22430 or FL1971. Moreover, although crystals were obtained for *Ls*-AChBP in the presence of FL1961, FL8454 and FL8561, there were no detectable electron densities for these fragments. In total, five structures of *Ls*-AChBP in complex with a ligand were successfully solved (Fig. 8, Table 2 and Fig. S6, Tables S1, S3).

**Table 2:** Overview of biosensor-based methods used to characterize interactions between ligands and *Ls*-AChBP and outcomes from X-ray crystallization experiments for the ligand-*Ls*-AChBP complexes. Some already published data are referenced. Estimated *K*_D_ values are provided when available

Compound	Biosensor data					X-ray crystallography					
	SPR 2° effects *K*_D_^a^ (μM)	GCI *K*_D_^b^ (μM)	SAW *K*_D_^c^ (μM)	Characteristic ΔSHG^d^ (%)	Helix DLC^e^	X-ray structure (.PDB)	Space group	Resolution (Å)	# subunits	# occupied sites	Calculated hydrodynamic radius^f^ (Å)
Lobeline	Yes	0.054		<10@10 μM	−41	2BYS ^4^					
	0.2										
VUF6105	Yes		23	>90@250 μM	−30	8Q1T	*P*2_1_2_1_2_1_	2.0	10	5	50.4
VUF22430	Yes	1.3	4.1	>800@250 μM		No crystals	N/A	N/A	N/A	N/A	N/A
VUF24234	Yes	5	1.1	>70@250 μM	+40	8P22	*P*2_1_2_1_2_1_	2.1	10	8	49.8
FL1613	No			>70@250 μM		8P1E	*P*2_1_2_1_2_1_	2.1	10	2	51.2
FL1856	Yes	0.585		>40@250 μM		7NDP ^22^	*P*2_1_2_1_2_1_	2.0	10	8	50.0
FL1888	Yes	6.3		>60@250 μM		7NDV ^22^	*P*2_1_2_1_2_1_	1.7	10	5	50.6
FL1909	No					8P1F	*P*1 2_1_ 1	2.1	20	3	67.6
FL1961	Yes			Negative@250 μM		Crystals, no ED^g^	*P*2_1_2_1_2_1_	N/A	N/A	N/A	N/A
FL1971	Yes	0.284		>300@250 μM		No crystals	N/A	N/A	N/A	N/A	N/A
FL3044	No					8P11	*P*2_1_2_1_2_1_	1.9	10	5	49.6
FL8454	No					Crystals, no ED^g^	*P*2_1_2_1_2_1_	N/A	N/A	N/A	N/A
FL8561	No			>10@250 μM		Crystals, no ED^g^	*P*2_1_2_1_2_1_	N/A	N/A	N/A	N/A
	63										

a In Fig. 3 and Fig. S1 or ref. 23.

b In Fig. 4, or ref. 22.

c In Fig. 5.

d In Fig. 6.

e In Fig. 7.

f Predicted *via* HullRad.^35^

g No ED: no electron density observed for ligand.

The crystals belonged to space groups *P*2_1_2_1_2_1_, *P*12_1_1 or *C*121, and data up to 1.9–3.0 Å were used for integration and model refinement (Tables S1 and S3). Additional density was found in all structures at the interface between the subunits in the orthosteric agonist and antagonist binding site, next to loop C in which the respective ligands could be modelled (Fig. 8). The unit cell was comprised of two or four pentamers of *Ls*-AChBP in the presence of FL1613, FL1856, FL1888, FL3044, VUF24234 and VUF6105. However, the number of binding sites in which additional electron density was observed varied.

The electron density in the binding site region was generally poor, indicating that compounds may have had several binding modes in the site. Although conformational changes in loop C were observed, there was no correlation between the binding of the ligand and the movement of loop C. Amino acid residues 175–180 in the protein could not be modelled due to poor electron density in this region. This indicates that it is a dynamic part of the protein.

### Structural comparison of ligand induced conformational changes

**Fig. 9:**
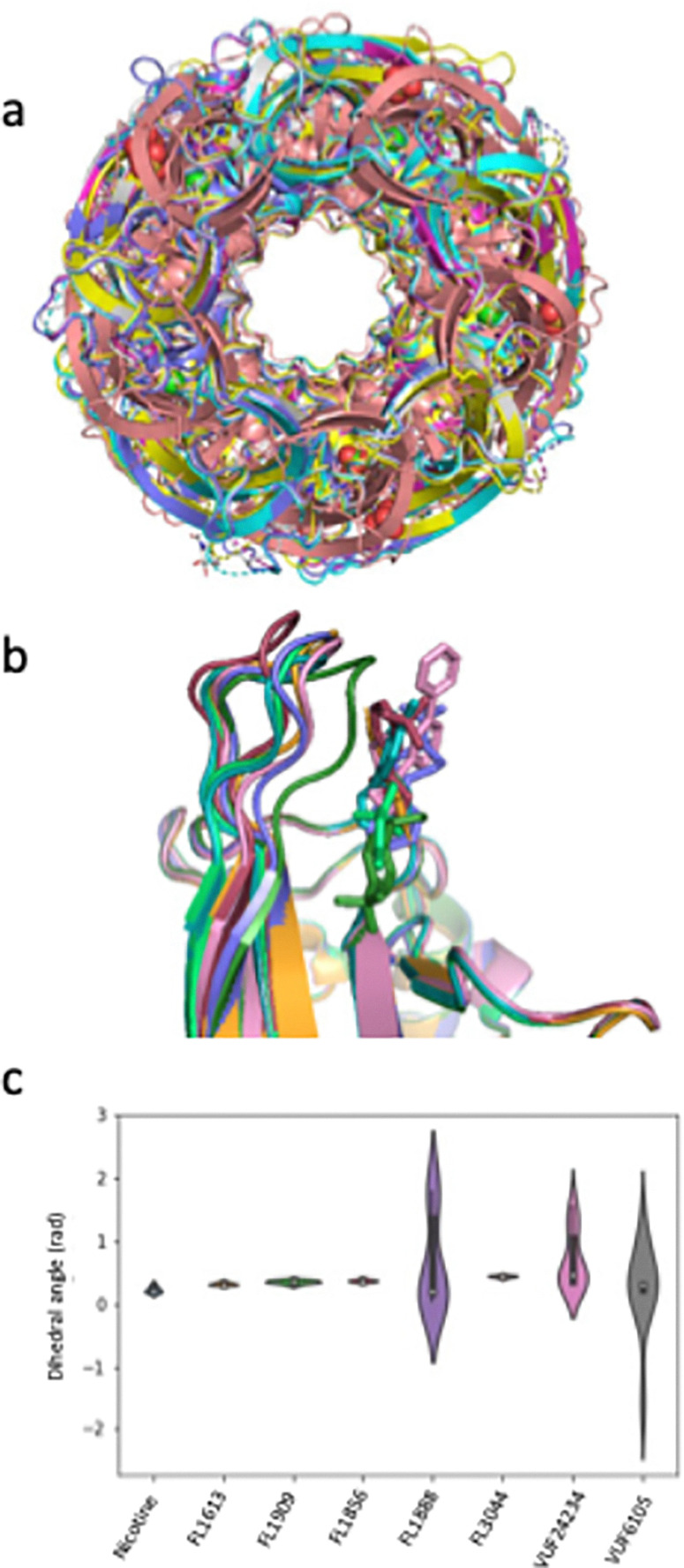
Comparison of conformational changes occurring in *Ls*-AChBP upon binding of ligands. (a) Superimposed structures of globally aligned apo- and *Ls*-AChBP-ligand complexes. (b) Loop C region of the crystal structures of *Ls*-AChBP bound to FL1888 (forest green), VUF24234 (pink), VUF6105 (orange), FL1613 (raspberry), FL1909 (slate blue), FL1856 (teal) and FL3044 (lime green). (c) Violin plot of the dihedral angle over amino acid residues Y204, C206, C207 and P208, located in loop C.

A computational alignment and superimposition of AChBP taken from the protein–ligand complexes and the apo structure showed that there were no large global conformational changes in any of the structures upon ligand binding (Fig. 9(a)). However, there were small changes in the loop C region of the crystal structures of Ls-AChBP (Fig. 9(b)). When comparing the protomers in complex with a ligand we observed that loop C moves differently upon binding of different compounds. Notably, binding of FL1888, VUF24234 and VUF6105 results in a movement of the loop closer to the compound, while binding of FL1856, FL3044, FL1613 and FL1909 does not have the same effect. The magnitude of the changes in loop C were quantified as the dihedral angle over amino acid residues Y204, C206, C207 and P208, located in loop C, and visualised in a violin plot (Fig. 9(c)). The plot indicates that binding of nicotine, FL1613, FL1909, FL1856 and FL3044 do not result in any significant conformational changes in this region, while binding of FL1888, VUF24234 and VUF6105 do.

## Discussion

It has herein been demonstrated that several types of biosensors can be useful for the detection and characterisation of ligand induced conformational changes in AChBP, even when interactions are rapid, weak and structural changes small. Although AChBP is a well-established model system for LGICs, the information that has been possible to obtain by other biophysical methods has been limited. X-ray crystallography has been the standard method for establishing binding sites and binding modes for ligands. However, we have experienced that for AChBP and other proteins with structurally flexible or disordered regions it has been challenging to obtain any crystals (with or without ligand) or detect electron densities for ligands in the structures, although biosensor experiments have been useful for identifying and characterising the interactions, *e.g.* SMYD3.^26^ This may be aggravated by the often weak and rapid interactions of the small fragments or peptides we have used in our work since they may have multiple binding orientations and low occupancies, resulting in heterogeneous complexes that are suboptimal for crystallisation or incomplete electron densities. Although new methods addressing such issues are being developed for X-ray crystallography,^27^ biosensor-based approaches are attractive complements. They have a unique potential to discriminate the binding of ligands from the induced structural effects and can thereby provide important mechanistic and kinetic information on the dynamics of LGIC function and regulation and guide ligand discovery and optimisation for therapeutic applications.

The groundbreaking SPR biosensor technology is used for a wide range of applications involving time-resolved analysis of molecular interactions. Although complex interactions, such as ligand induced conformational changes, can be studied, few studies focus on the detection and characterisation of these. This is partly due to the risk that observed complexities in sensorgrams are due to artifacts or suboptimal experiments. In our initial studies using this technology for analysis of ligand interactions with AChBP, complex SPR sensorgrams were obtained for a number of ligands, and were interpreted as indicative of ligand-induced conformational changes, not to unspecific binding or instrument related artifacts.^10^ The possibility of detecting such changes is rationalized as a collapse and subsequent recovery in the dextran matrix as a direct result of the LGIC twisting or breathing motions, thereby influencing the refractive index of the sensor surface which is monitored by the instrument.

Here we have explored the newer SAW, SHG and switchSENSE biosensor technologies which all have potential for direct detection of conformational changes upon ligand binding. They vary in their detection systems and have quite different experimental setups. This influences the information that can be obtained as well as practical aspects such as throughput, material consumption and cost. Although neither the SAW nor SHG technologies are commercially available at present, the current data show that these technological principles are useful.

For comparison, we also used a GCI biosensor. It is very similar to SPR biosensors, but it has a slightly different sensor surface, detection principle and microfluidic system.^28–30^ It is known to have a superior time resolution, allowing estimation of the rate constants for very rapid interactions.^26,28^ But we hypothesise that its lower sensitivity to conformational changes is primarily due to matrix effects on the sensor surface.

We have previously generated GCI data for fragments FL1856, FL1888 and FL1971 interacting with AChBP.^22^ Kinetic rate constants and *K*_D_-values could be estimated although the sensorgrams for FL1888 had distortions indicative of conformational changes. The GCI sensorgrams for the VUF-compounds tested here showed similar minor distortions. The sensorgrams for the reference compound lobeline did not appear to be distorted and were used for global, non-linear regression analysis and estimation of rate constants and *K*_D_-values. However, the *K*_D_-value determined using steady-state report points was slightly different, revealing that a 1 : 1 model is suboptimal and that lobeline also had a more complex interaction. The data for the VUF-compounds were clearly too complex for a global analysis of sensorgrams and only a steady-state analysis was therefore used for estimation of *K*_D_-values. In contrast, a steady-state analysis of the SPR biosensor data was not meaningful since there was no reliable steady state. Instead, the SPR biosensor data provided useful qualitative information about the complexity of the interactions. Since this was not evident in the GCI data, it would typically be overlooked. Clearly, the technologies can be complementary.

To further explore the ability of the ligands of interest in this study to induce conformational changes in AChBP, we used an SHG-based biosensor. We have previously shown that this technology has the sensitivity and throughput required for screening, detection and characterisation of small organic molecules inducing conformational changes in AChBP.^22^ This equilibrium-based technology requires labelling of the target protein with a second-harmonic active (SH-active) dye probe.^31^ Ligand-induced conformational changes which result in a net dye movement are quantified as a change in the SHG signal (ΔSHG). Compounds which do not induce a conformational change in the labelled protein will not result in a signal change. The detection is independent of the size of the target and ligand, and can be applied (and is ideally suited) to large proteins such as the AChBP pentamer (125 kDa). Although concentration series of ligands are analysed, it is not suitable for determination of equilibrium constants as there are multiple factors contributing to the magnitude of the signal. An advantage is that the conformational sensitivity of the surface of the protein *vs.* a ligand can be probed by immobilising the SHG label in specific positions. An advantage of this technology is that it has the capability of providing structural information. By labelling the protein in several positions it can potentially distinguish different binding modes and provide a more complete conformational landscape of the target protein, as well as resolve predicted allosteric binding modes.

The SHG biosensor experiments showed that all ligands analysed induce conformational changes in essentially all variants of AChBP (for details regarding the position of labels, see ref. 22) although the signal magnitudes and selectivity profiles were slightly different. This is consistent with the observation that these ligands all interact in the same region of the protein, *i.e.* the subunit interface close to loop C. However, based on these data it is not known if the observed conformational changes are local rearrangements, *e.g.* resulting from movement of loop C as a consequence of accommodating the ligand, or if they are larger global conformational changes such as pore opening, triggered by ligand binding.

The SAW biosensor is also an interesting technology for direct detection of ligand induced conformational changes in proteins. It is based on piezoelectric changes in a substrate that allows an electrical signal to be converted into an acoustic one. Changes in mass result in a shift in the phase of the acoustic wave, whereas a variation of the flexibility of the molecule is reflected in shifts in its amplitude.^32^ Although the data look similar to that from SPR and GCI biosensors, it is only phase data that is directly comparable. The amplitude data are unique and reflect the conformational change.

The *K*_D_ values estimated from the preliminary data set generated herein are very rough approximations due to suboptimal experiments. In principle *K*_D_ values can be estimated from phase data, but the interactions did not reach steady state, which would be required for reliable estimates. Despite these caveats, estimated affinities were in the μM range, as for other methods. Although the amplitude data have a signal *vs.* concentration dependency, its theoretical interpretation is hampered by the fact that it is correlated with the magnitude of the conformational change rather than the binding event.

Nevertheless, it was noted that similar *K*_D_-values were obtained from both types of signals. Differences were also seen for the different compounds and their interactions with *Ls*- and *Ac*-AChBP, showing that this technology can in principle be used to confirm and quantify both binding and conformational changes, and provide data suited for structure–activity relationships. Unfortunately, the instrument available for the project was not sufficiently robust for a larger series of experiments and it was later discontinued as a commercial product. Despite these limitations, these experiments represent a unique proof-of-principle for this technology.

The switchSENSE biosensor detects ligand-induced conformational changes in proteins by monitoring changes in the hydrodynamic friction between the protein (with and without added ligand) and the solvent (Fig. S5a).^33,34^ Proteins are tethered to a chip surface *via* rigid DNA nanolevers which are electrically actuated to oscillate at high frequencies. The motion of the DNA nanolevers is traced in real time by the fluorescence of an integrated fluorophore, which is quenched by the chip surface in a distance dependent manner. Ligand-induced conformational changes of tethered proteins that alter the protein's hydrodynamic friction will result in a change in motion speed of the entire nanolever–protein complex during switching. A ligand-induced expansion in the protein will cause the entire protein–DNA nanolever complex to experience a higher degree of friction and thus move more slowly into the upright position (Fig. S5b). Conversely, the opposite is true for a ligand-induced compaction in the protein.

A unique set of data was generated using the switchSENSE biosensor. It showed that two of the compounds (lobeline and VUF6106) induced a compaction of the AChBP structure, while a third compound (VUF24234) resulted in an expansion. This indicates that these compounds have very different binding modes and structural consequences of binding. This does not match the predicted hydrodynamic radii for the complexes, which therefore seem to be unreliable for AChBP complexes (Table 2).

The crystallisation of AChBP in complex with the compounds was important to enable a correlation of the observations from biosensor experiments with actual structural changes. Unfortunately, the co-crystallisation of the ligands did not result in observable electron density for all ligands in all binding pockets. This may be due to low ligand affinities, incompatibilities with crystal packing or high flexibility of the binding interface. Nevertheless, the structures obtained were scrutinised to identify differences in binding modes and if conformational changes are local rearrangements or larger global conformational changes.

The violin plot revealed that conformational changes in loop C occurred to a higher degree in the AChBP structures with compounds FL1888, VUF24234 and VUF6105 than in the other structures. This correlated with the results from the SPR, SAW and SHG biosensor experiments, which all indicated conformational changes in AChBP upon binding of these compounds. Interestingly, the ligand that resulted in distorted SPR and GCI biosensor sensorgrams and the largest ΔSHG signals (VUF22430) did not give any crystals (and is therefore not in the violin plot). This suggests that the magnitude of the structural change interfered with the crystallisation. It could also be that the crystal packing does not permit the conformational change needed to bind these compounds.

An overview of the data for a variety of compounds analysed is provided in Table 2. It shows that data providing various types of information about ligands interacting with AChBP could be generated with five different types of biosensors and X-ray crystallography. The table is incomplete due to limited access to some compounds and instruments, but some data are missing since the method did not work. Notably, X-ray crystallography was not successful for many of the ligands and data could not be obtained for a ligand that interacted with DNA on the switchSENSE sensor surface.

A complicating factor when analysing interactions that are more complex than a simple 1 : 1 Langmuir interaction is that it is often difficult to establish the exact interaction mechanism. For example, in the case of the SPR biosensor data presented here, it would not be meaningful to globally fit any model to the sensorgrams, nor fit a Langmuir model to report points taken at the end of the injection, although they appear to represent steady state. Similarly, GCI biosensor data may be influenced by complex effects, even if the data appear to be well described by a simple interaction model. By exploring relevant interaction models for global non-linear regression analysis of the initial data, followed by suitable experimental controls and generation of high-quality datasets, kinetic parameters may be estimated. Although this could provide valuable knowledge about the interaction and can be used for structure–activity relationship analyses, such data do not provide any structural information regarding the magnitude or nature of the induced structural changes. A reliable method for structural analysis of the dynamics of ligand-induced structural changes that complements X-ray crystallography would consequently be of great interest for this field.

## Experimental

### Protein expression & cys mutants

Proteins were expressed and purified as previously described.^22^

### Surface plasmon resonance biosensor analysis

All experiments were performed with a Biacore T200 surface plasmon resonance biosensor instrument (GE healthcare). Proteins were immobilized on CM5 series S sensor chips. Consumables were obtained from GE Healthcare. All solutions were freshly prepared, degassed, and filtered. The proteins were buffer exchanged using a Amicon Ultra 0.5 mL centrifugal filter (Merck) to 137 mM NaCl, 2.7 mM KCl, and 10 mM phosphate buffer pH 7.4 and diluted to 1 mg mL^−1^. The protein immobilization was run in 137 mM NaCl, 2.7 mM KCl, and 10 mM phosphate buffer pH 7.4 at a flow rate of 10 μL min ^−1^ at 25 °C. The matrix of the sensor chip was activated by injecting a mixture of 0.1 M *N*-hydroxysuccinimide (NHS) and 0.4 M 1-ethyl-3-(3-(dimethylamino)propyl) carbodiimidehydrochloride (EDC) over all flow channels for 420 seconds. Subsequently, protein (0.05–0.01 mg mL^−1^) in a 10 mM NaAc solution (pH 5.0 for *Ls*-AChBP and pH 5.5 for *Ac*-AChBP) was injected for 300 s. Unreacted activated groups of the dextran matrix were deactivated by injection of ethanolamine-HCl (1 M) for 420 s. The final immobilization levels were between 3000 RU and 8000 RU.

All compounds were dissolved in DMSO (stock solutions of 10 mM) and diluted in 137 mM NaCl, 2.7 mM KCl, and 10 mM phosphate buffer pH 7.4, 0.05% Tween-20 (v/v), and 5% DMSO (v/v). Suitable concentration series were determined for the reference compounds according to already known *K*_D_-values. The investigated compounds highest concentration injected was 100 μM. A 7-point concentration series of each compound was measured in multicycle experiments. Compounds were injected for 60 s and the dissociation was monitored for 300 s at 50 μL min^−1^. Each cycle includes a regeneration step where a solution of 137 mM NaCl, 2,7 mM KCl, and 10 mM phosphate buffer pH 7.4, 0.05% Tween-20 (v/v), and 10% DMSO (v/v) was injected for 10 s at 100 μL min^−1^ over all flow channels, followed by an extra wash of the injection system with 50% DMSO (v/v). All titrations were run at 25 °C at a flow rate of 50 μL min^−1^. Data analyses were performed with Biacore T200 evaluation software. Signals from reference surfaces and blank injections were subtracted from the observed signals (double referencing). DMSO corrections were performed. The affinities of the reference compounds were determined by fitting a Langmuir binding equation to steady state binding signals at different concentrations.

### Surface acoustic wave biosensor analysis

All experiments were performed with a NanoTemper Seismos biosensor instrument (NanoTemper Technologies Gmbh).††The Seismos SAW biosensor system has been discontinued. Proteins were immobilized on 3D CM-Dextran sensor chips. Consumables were obtained from NanoTemper Technologies. All solutions were freshly prepared, degassed, and filtered.

The protein immobilization was run in 137 mM NaCl, 2.7 mM KCl, and 10 mM phosphate buffer pH 7.4 at a flow rate of 10 μL min^−1^ at 25 °C. The matrix of the sensor chip was activated by injecting a mixture of 0.1 M *N*-hydroxysuccinimide (NHS) and 0.4 M 1-ethyl-3-(3-(dimethylamino)propyl) carbodiimidehydrochloride (EDC) over all flow channels for 420 seconds. Subsequently, protein (0.05–0.01 mg mL^−1^) in a 10 mM NaAc solution (pH 5.0 for *Ls*-AChBP and pH 5.5 for *Ac*-AChBP) was injected for 300 s. Unreacted activated groups of the dextran matrix were deactivated by injection of ethanolamine–HCl (1 M, pH 8.5) for 420 s. The final immobilization levels were between 3000 RU and 5000 RU.

All compounds were dissolved in DMSO (stock solutions of 10 mM) and diluted in 137 mM NaCl, 2.7 mM KCl, and 10 mM phosphate buffer pH 7.4, 0.05% Tween-20 (v/v), and 5% DMSO (v/v). Suitable concentration series were determined for the reference compounds according to already known *K*_D_ values. The highest concentration of investigated compound injected was 5–100 μM. A 12-point concentration series of each compound was measured in multicycle experiments. Compounds were injected for 60 s and the dissociation was monitored for 500 s at 50 μL min^−1^. Each cycle includes a regeneration step where a solution of 137 mM NaCl, 2.7 mM KCl, and 10 mM phosphate buffer pH 7.4, 0.05% Tween-20 (v/v), and 10% DMSO (v/v) was injected for 10 s at 100 μL min^−1^ over all flow channels, followed by an extra wash of the injection system with 50% DMSO (v/v). All titrations were run at 25 °C at a flow speed of 50 μL min^−1^. Data analyses were performed with Ligand Tracer (Ridgeview Instruments AB, Uppsala Sweden) evaluation software. Sensorgrams were double referenced, *i.e.* signals from reference surfaces and blank injections were subtracted from the observed signals. DMSO corrections were performed. The affinity of the reference compounds was determined by fitting a Langmuir binding equation to steady state binding signals at different concentrations.

### Grating coupled interferometry biosensor analysis

All interaction kinetic experiments were conducted with a GCI, flow-based biosensor (WAVEdelta, Creoptix AG). The analysis temperature and running buffer composition, if not otherwise stated, were 25 °C with PBS-P+ buffer (20 mM phosphate buffer pH 7.4, 2.7 mM KCl, 137 μM NaCl, 0.05% Surfactant P20, Cytiva) supplemented with 1% DMSO (running buffer). The GCI data referencing and analysis were performed using WAVEcontrol software (Creoptix AG). AChBP was immobilized on a PCH WAVEchip (Creoptix AG) on the WAVEdelta. Sensor chips were conditioned using injections of borate buffer (10 mM sodium tetraborate pH 8.5, 1 M NaCl). The target protein (ligand) was diluted to the desired concentration in sodium acetate (10 mM, pH 5.0) depending on the required immobilization density. The sensor chip was functionalized for 420 s with EDC and NHS (Cytiva) and the target protein was amine-coupled to a final protein immobilization level of 6000 pg mm^−2^, using an injection time of 400 s and a flow rate of 10 μL min^−1^. After immobilization, the surface was deactivated with ethanolamine–HCl (1.0 M, pH 8.5) for 420 s.

Kinetic measurements for AChBP controls and fragments were performed with a two-fold serial dilution starting at 250 μM for each compound. Solvent correction was performed ranging from 0–2% DMSO. Blank samples of the running buffer, 1× PBS-P+ buffer or HBS-P+ buffer (0.01 M HEPES pH 7.4, 0.15 M NaCl and 0.05% v/v surfactant P20) both containing 1% DMSO, were injected during the measurements every fifth cycle. Samples were injected over the immobilized surface and a reference channel. The sensorgrams were solvent corrected and double-referenced (subtraction of the signal from reference channel and blank injections). Kinetic fitting was performed with WAVEcontrol software (Creoptix AG) with a suitable interaction model.

### Second harmonic generation biosensor analysis

Experiments were performed using the Biodesy Delta SHG system.‡‡The Biodesy Delta SHG biosensor system has been discontinued. Supported lipid bilayers containing Ni-NTA were prepared according to the manufacturer's instructions (Biodesy, Inc.) and were formed by fusion to the well surface of 384-well Biodesy plates. AChBP-SHG1 was tethered to the lipid bilayer membrane at a concentration between 0.25 and 1 mM depending on the experiment, in AChBP assay buffer PBS-P + (20 mM phosphate buffer pH 7.4 2.7 mM KCl, 137 mM NaCl, 0.05% Tween 20) and incubated overnight at 4 °C. After it was tethered, wells were washed with assay buffer to remove unbound protein.

Ligand injections and SHG detection were carried out on the Biodesy Delta as follows: after reading the baseline SHG signal, 20 mL of ligand at 2 times the desired concentration was injected onto 20 mL of solution volume. The SHG signal change was defined as the percentage change in SHG intensity, ΔSHG (%), and calculated as ((*I*_*t*_ − *I*_*t*0_)/*I*_*t*0_) × 100, where *I*_*t*_ is the SHG intensity at time *t* and *I*_*t*0_ is the SHG baseline intensity before injection.

*K*_D_ values for control compounds were determined using SHG data points from a concentration series. The data were fitted by non-linear regression using Prism (GraphPad Software, San Diego, CA, USA) and an equation specific to SHG-derived CRCs.^31^

### switchSENSE biosensor analysis

*Ls*-AChBP was conjugated to the Dynamic Biosensors ligand strand with the amine coupling kit HK-NHS-1 (Dynamic Biosensors GmbH) according to the manufacturer's instructions. The DNA–protein conjugate was purified from non-conjugated oligonucleotide and free protein by a proFIRE chromatography system (Dynamic Biosensors GmbH). The fractions from the first conjugate peak were collected and buffer exchanged to PE140 (10 mM Na_2_HPO_4_/NaH_2_PO_4_, pH 7.4, 140 mM NaCl, 50 μM EDTA, 50 μM EGTA, 0.05% Tween-20). DNA–protein conjugates were diluted to 1 μM, aliquoted, snap frozen, and stored at −80 °C for future use.

The purified *Ls*-AChBP–DNA conjugate was hybridized to DNA origami nanolevers according to the manufacturer instructions (HK-ORM-1, Dynamic Biosensors GmbH). In short, the protein-conjugate was diluted to 250 nM in a phosphate-based origami buffer and mixed with 20 nM origami nanolever 1 in a 1 : 1 ratio (v/v). Similarly, the ligand-free strand was diluted to 250 nM and mixed with 20 nM origami nanolever 2 in a 1 : 1 ratio (v/v). These two mixtures were separately incubated for 2 hours at 25 °C and 600 rpm, while being protected from light. The mixtures were then mixed at a 1 : 1 ratio (v/v) and were ready to use for switchSENSE conformational change analysis.

A heliX^+^ biosensor system, standard adapter chips and the heliOS ‘conformational change scouting – origami’ method were used (dynamic biosensors GmbH) for conformational change experiments. Lobeline was dissolved in pure water to 10 mM and then diluted to 100 μM in PE140 for analyte injections. PE140 buffer was used as the running buffer and blank injection. VUF6105 and VUF24234 were dissolved in 100% DMSO to 100 mM and then diluted in PE140 to 100 μM. PE140 + 0.1%DMSO buffer was used as the running buffer and blank injection. Experimental components were loaded into the heliX+ biosensor according to the assay instructions in the heliOS software. Automated data analysis was performed using the heliOS software with a dynamics integration window set to 0–350 μs. Dynamic lag change (DLC) values were calculated as in eqn (1) from triplicate repeats of switching cycles before and after ligand injection.1
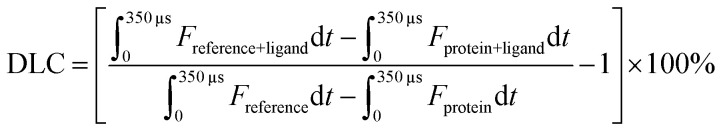


### Computational analysis

MOE 2016.0802 (Chemical Computing Group, ULC, 910-1010 Sherbrooke St. W., Montreal, QC H3A 2R7, Canada) was used to align and superimpose the protein complexes with the apo structure of the corresponding AChBP. The alignment was performed using the mixed sets of structural and sequence-only information data in order to determine the correspondences between residues of the different pentameric complexes. The Protein Structure Superposition application was used to superpose the 3D protein structures.

The all-against-all Cα-RMSD distances between apo and holo structures were calculated respectively for the complete pentamer and for the M-loop. The RMSDs are displayed in the colour-coded box. The colour legend is shown on the right side. The colour scale is clamped at 4.0 Ångström; all values above 4.0 are coloured dark red in the plot.

### Protein crystallization, data collection and model building

*Ls*-AChBP at 10 mg mL^−1^ in storage buffer (20 mM HEPES, 137 mM NaCl, 2.7 mM KCl, pH 7.4) was incubated with compound dissolved in DMSO, resulting in a final concentration of 2.5 mM compound and 5% DMSO. The drops of 2 μL contained a 1 : 1 ratio of protein–compound mix and reservoir solution (100 mM citric acid at pH 4.8–5.2 and 1.5–2 M ammonium sulphate). The crystallization experiments, performed in a hanging drop vapour diffusion setup at room temperature, resulted in crystals of various morphologies forming after 1–2 weeks. The crystals were cryo-protected in a reservoir solution supplemented with 20% glycerol before snap-freezing in liquid nitrogen. Diffraction data were collected at the Diamond Light Source (Oxford, UK) I04 beamline and the MAXIV (Lund, Sweden) BioMAX beamline. Indexing, merging and scaling was done using XDS,^36^ XSCALE,^37^ and XDSCONVERT.^37^ Molecular replacement was carried out with PhaserMR^38^ with the structure deposited with PDB accession code 1UW6^15^ as the search model. The ligand dictionaries were created using AceDRG.^39^ Model building and structure refinement were carried out using Coot^40^ and REFMAC5,^41^ respectively. Figures were prepared with the PyMOL Molecular Graphics System (Schrödinger, LLC). The hydrodynamic radii of the complexes were calculated using HullRad.^35^

## Conclusions

This study has highlighted that the mechanism of LGIC function is finetuned and can respond to interactions with small regulators whose function is dependent on both binding and a binding mode that triggers a specific conformational change that can translate into a large structural rearrangement in a protein. The combined biosensor-based and structural data obtained herein illustrate that different types of biosensor-based methods can be used to study the process of ligand binding, which is distinct from the structural rearrangements that result from the binding. By using methods that can provide information on both processes, it is expected that we can better understand regulation of structurally dynamic proteins and develop therapeutics that can specifically modulate their function. This is relevant not only for LGICs and other ion channel receptors, but also intracellular receptors and enzymes, for example.

X-ray crystallography provides structures for the native free protein and the protein in complex with the ligand, but as snapshots without showing the interaction dynamics. The biosensor data are clearly complementary. Many of the biosensors showed qualitative differences in the data for different compounds, indicating that they interacted with different sites or in different binding modes. This might be more informative than the affinities or kinetic values that might be extracted from a quantitative analysis and kinetic parameters.

Our interest has been to establish methods that can further our understanding of the complex molecular interactions as well as that can be used to identify and guide ligand optimisation for therapeutic purposes. We were looking for methods that could provide complementary information to X-ray crystallography. Clearly, there are several types of biosensor technologies that can be useful, although some are still in their early stages of development. High quality datasets showing how they can be implemented in projects are often lacking, making commercialisation challenging. We expect that this study will encourage the innovation and further development of new methods for the field.

## Author contributions

U. H. D., I. J. P. E., E. A. F. and P. B. conceived the project. E. A. F. and D. K. produced the proteins. I. J. P. E. provided the LGIC ligands. P. B. performed the original SPR biosensor experiments and E. A. F. performed SPR, SHG, SAW and GCI biosensor experiments. D. K. and P. P. P. carried out the switchSENSE experiments. E. A. F., P. B., D. C, D. D., S. H. and B. A. L. carried out the crystallographic studies. U. H. D. supervised the project. E. A. F, D. C. and U. H. D. wrote the manuscript.

## Conflicts of interest

Edward Fitzgerald is an employee of Malvern Panalytical, provider of the Wave GCI biosensor. Pablo Porragas Paseiro is employed as an early-stage researcher at Dynamic Biosensors GmbH, provider of the switchSENSE biosensor. The other authors have no conflicts to declare.

## Supplementary Material

CB-006-D5CB00041F-s001

## Data Availability

Most data are provided in the manuscript and SI. The SI contains figures with additional experimental data from SPR, GCI, SAW, SGH and switchSENSE biosensors, a description of the detection principle for switchSENSE experiments and additional structures of AChBP in complex with ligands. Tables with X-ray crystallography data collection and refinement statistics are also provided. See DOI: https://doi.org/10.1039/d5cb00041f Additional data are available upon request. Crystallographic data for compounds FL1909, FL3044, FL1613, VUF24234 and VUF6105 have been deposited at the PDB under accession numbers 8P1F, 8P11, 8P1E, 8P22 and 9SG3, respectively.
