# Supplementary material for: Detection and characterisation of ligand-induced conformational changes in acetylcholine binding proteins using biosensors and X-ray crystallography
Source: RSC Chem Biol. 2025 Aug 13;6(10):1625–39. doi: 10.1039/d5cb00041f (PMC12394895; doi:10.1039/d5cb00041f)
Supplement: CB-006-D5CB00041F-s001 [file CB-006-D5CB00041F-s001.pdf]

Supplementary Information (SI) for RSC Chemical Biology.
This journal is © The Royal Society of Chemistry 2025

Supplementary material

Detection and characterisation of ligand-induced conformational changes in proteins using biosensors and X-ray crystallography

Edward A. FitzGerald,[1, 2] Daniela Cederfelt,[1] Daria Kovryzhenko,[1] Pierre Boronat,[3] Bjarte Aarmo Lund,[4] Doreen Dobritzsch,[1] Sven Hennig,[5,6] Pablo Porragas Paseiro,[7] Iwan J.P. de Esch,[3] and U. Helena Danielson*,[1,6]

[1]*Department of Chemistry - BMC, Uppsala University, Sweden.*
[2]*Beactica Therapeutics AB, Virdings allé 2, Uppsala, Sweden.*
[3]*Division of Medicinal Chemistry, Amsterdam Institute of Molecular and Life Sciences (AIMMS), Vrije Universiteit Amsterdam, De Boelelaan 1108, 1081 HZ Amsterdam, The Netherlands.*
[4]*Department of Chemistry, UiT The Arctic University of Norway, Tromsø, Norway.*
[5]*Department of Chemistry and Pharmaceutical Sciences, Vrije Universiteit Amsterdam, De Boelelaan 1108, 1081 HZ Amsterdam, The Netherlands*
[6]*Amsterdam Institute of Molecular and Life Sciences, Vrije Universiteit Amsterdam, De Boelelaan 1108, 1081 HZ Amsterdam, The Netherlands*
[7]*Dynamic Biosensors GmbH, Perchtinger Str. 8/10, 81379 München, Germany*
[6]*Science for Life Laboratory, Uppsala University, Uppsala, Sweden.*
**Corresponding author. E-mail: helena.danielson@kemi.uu.se*

Table of Contents

Figure S1. SPR biosensor data 2
Figure S2. GCI biosensor data for lobeline 4
Figure S3. SAW biosensor data 5
Figure S4. SHG data 6
Figure S5. switchSENSE principle for detection of conformational changes and motion curves. ...7
Figure S6. Crystal structures of AChBP in complex with ligands 9
Table S1. Data collection and refinement statistics for crystal structures of AChBP and FL-compounds 10
Table S2. Data collection and refinement statistics for crystal structures of AChBP and VUF-compounds 11

Figure S1. SPR biosensor data

Examples of sensorgrams for fragment hits from an SPR biosensor screen of a fragment library against *Ls*-AcCBP.[17] Sensorgrams with single concentrations (250 μM): (a) FL1856, (b) FL1888, and concentration series up to 250 μM (left) and corresponding dose response plots (right): (c) FL3044, (d) FL1613, (e) FL1909, (f) FL8454, and (g) lobeline with best fitting lines and data from non-linear regression analysis using a heterogeneous ligand model (black traces) and a steady state analysis using a 1:1 interaction model.

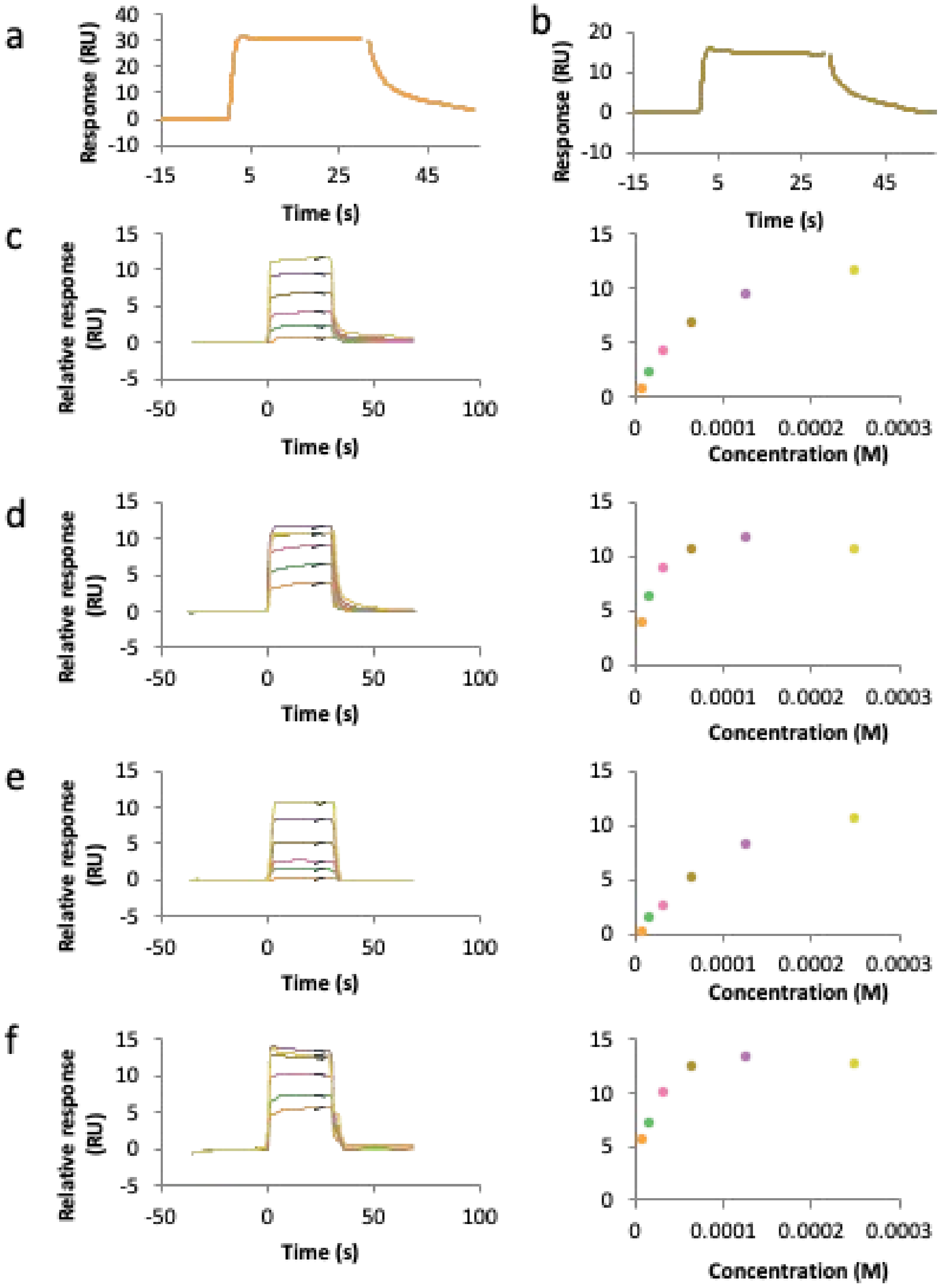

g

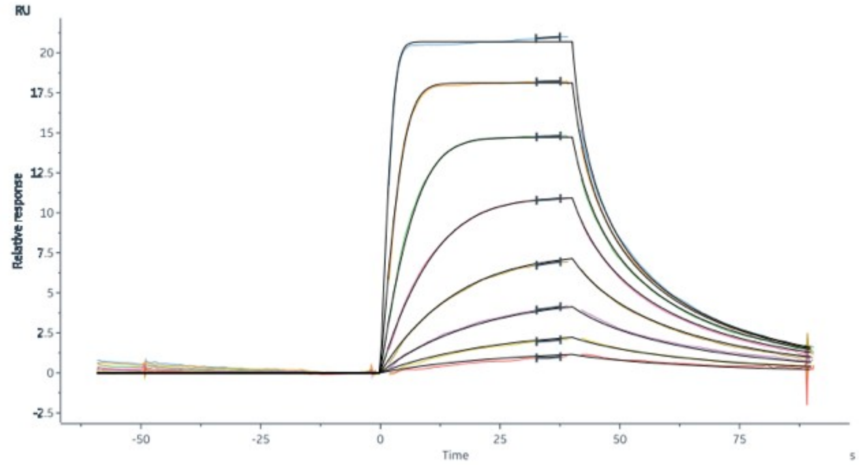

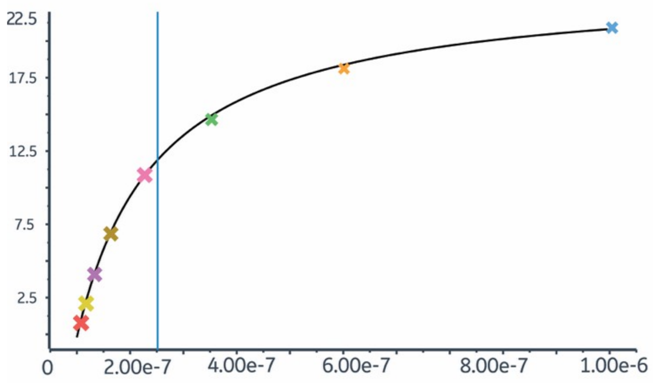

Concentration (M)

**Kinetic Evaluation (Heterogeneous Ligand Model)**		
R_{max1}	1.59E+01	RU
R_{max2}	8.51	RU
k_{a1}	2.13E+06	$M^{-1}s^{-1}$
k_{d1}	5.51E-01	s^{-1}
k_{a2}	1.43E+06	$M^{-1}s^{-1}$
k_{d2}	8.34E-02	s^{-1}
K_{D1}	258.7	nM
K_{D2}	58.3	nM
Sqrt(Chi2)	2.93E-02	pg/mm^2
**Equilibrium Analysis**		
R_{max}	6.4	RU
K_D	152	nM
Offset	-0.2	RU
Sqrt(Chi2)	1.46E-02	RU^2

Figure S2. GCI biosensor data for lobeline

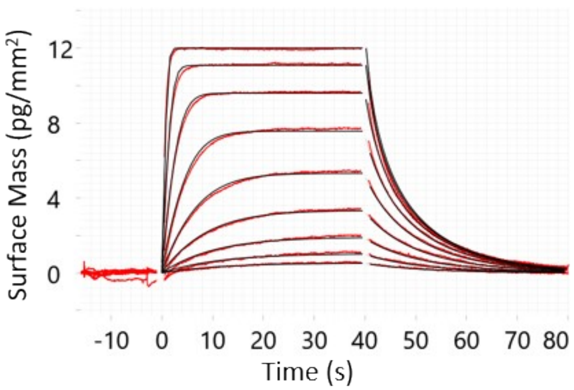

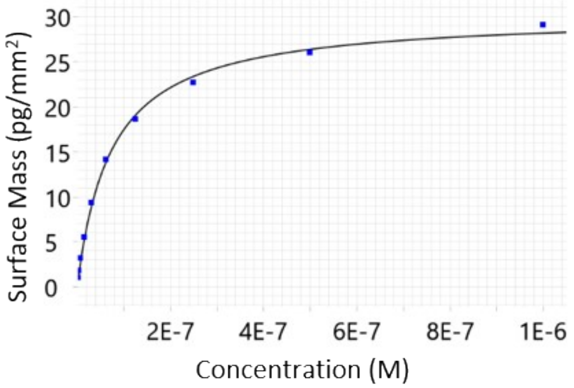

**Kinetic Evaluation (1:1 Model with Mass Transport)**		
R_{max}	26.207	pg/mm²
k_t	2.84E7	s^{-1}
k_a	1.31E7	$M^{-1}s^{-1}$
k_d	7.11E-1	s^{-1}
K_d	54.5	nM
Sqrt(Chi2)	0.274	pg/mm²
**Equilibrium Analysis**		
R_{max}	29.969	pg/mm²
K_D	79.2±17.9	nM
Offset	0.509	pg/mm²

Figure S3. SAW biosensor data

Phase and amplitude signals for compounds with both *Ls*-AChBP and *Ac*-AChBP, the data was used for estimation of equilibrium constants (Table 1).

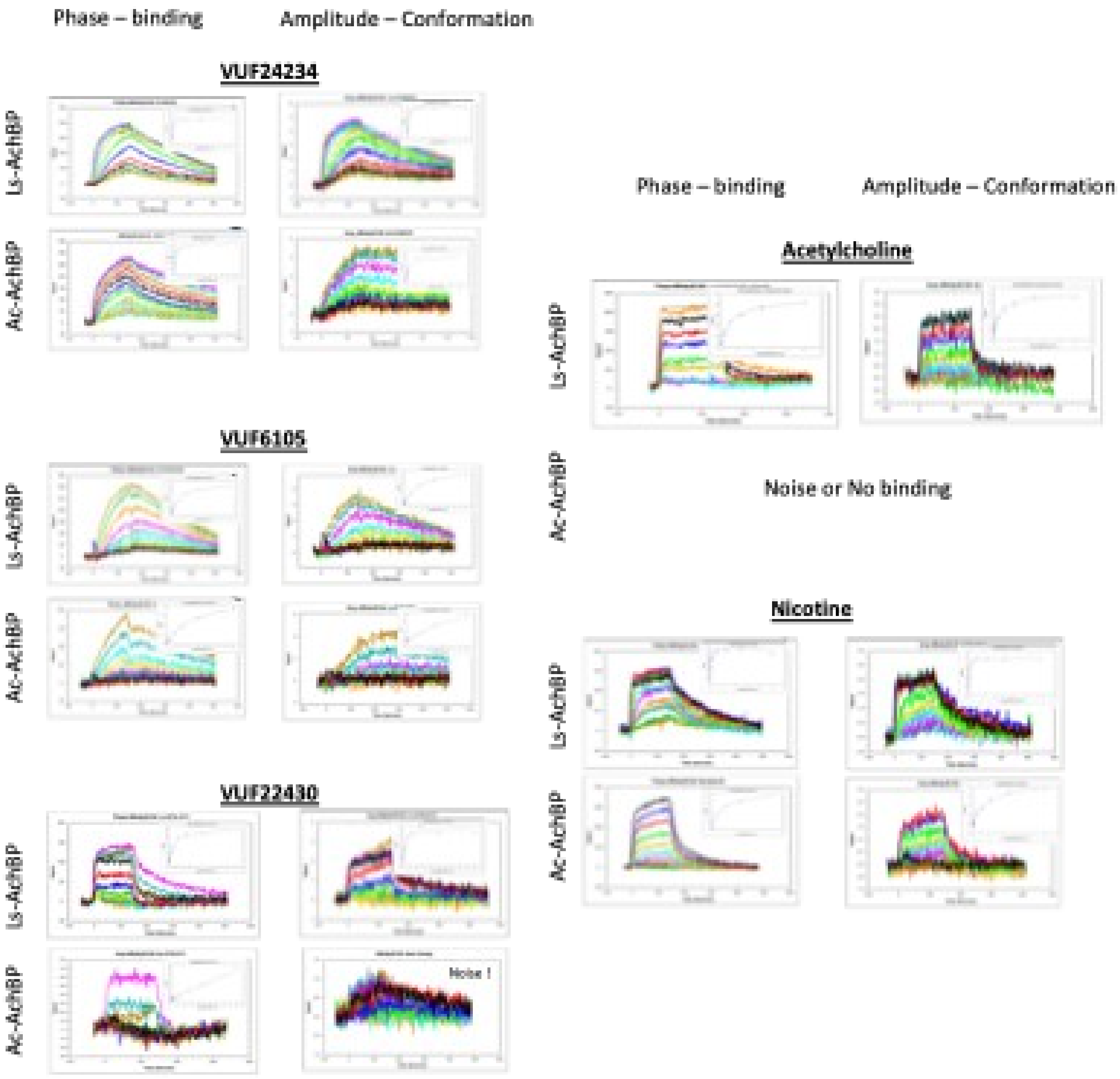

Figure S4. SHG data

Concentration response curves (CRC) and time course data.

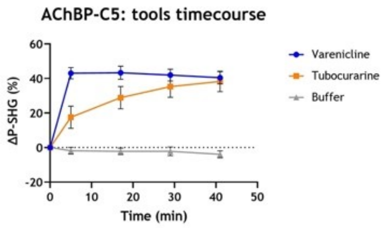

VUF22430

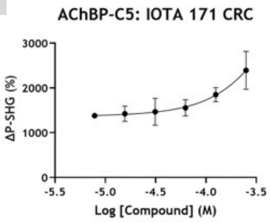

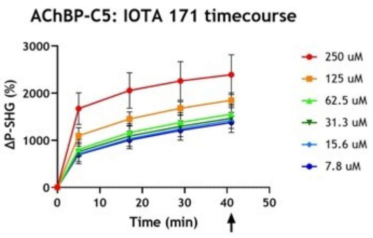

FL1971

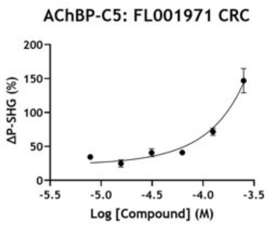

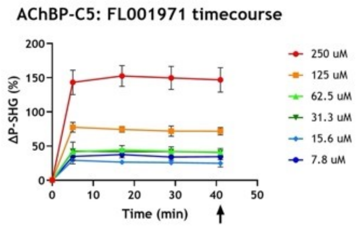

FL1856

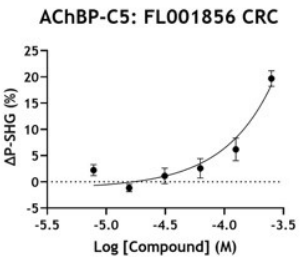

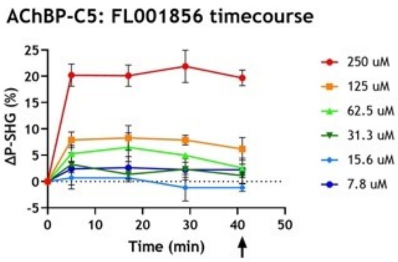

FL1888

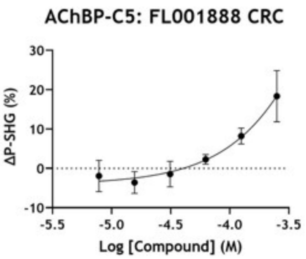

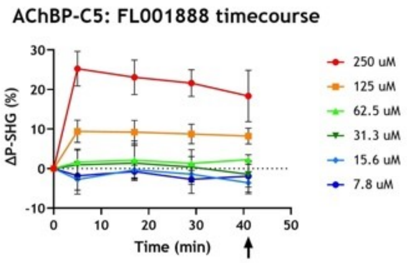

FL1961

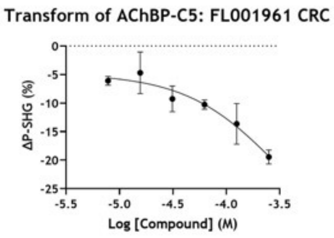

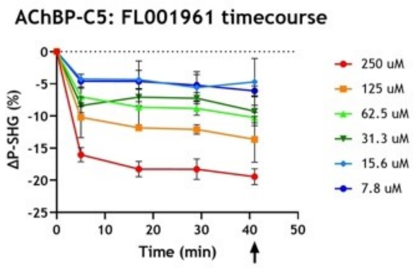

Figure S5. switchSENSE principle for detection of conformational changes and motion curves.

(a) Principle for sensor surface with protein attached to origami lever (left), reference lever (right, and fluorescence vs. time signals for different surfaces with and without added analyte (middle). (b) Principle for detection of protein compaction and expansion upon interaction with an analyte. (c) Motion curves for all tested compounds and corresponding blanks.

a

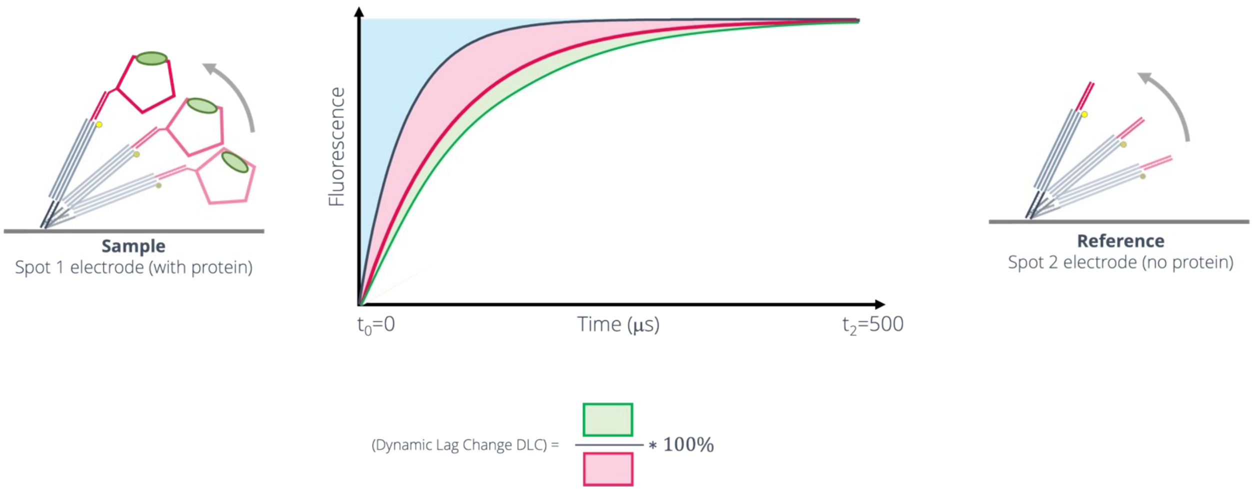

b

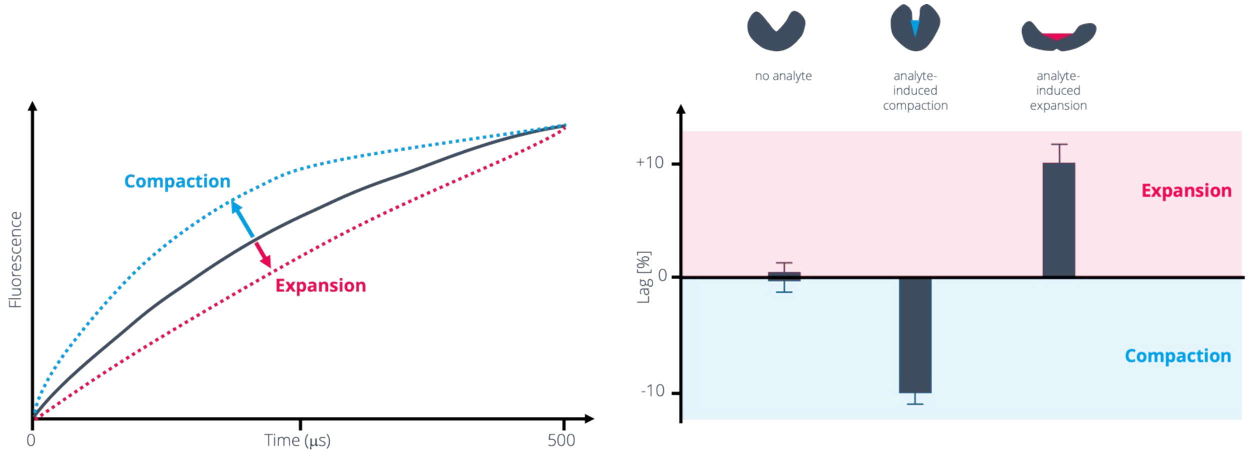

c

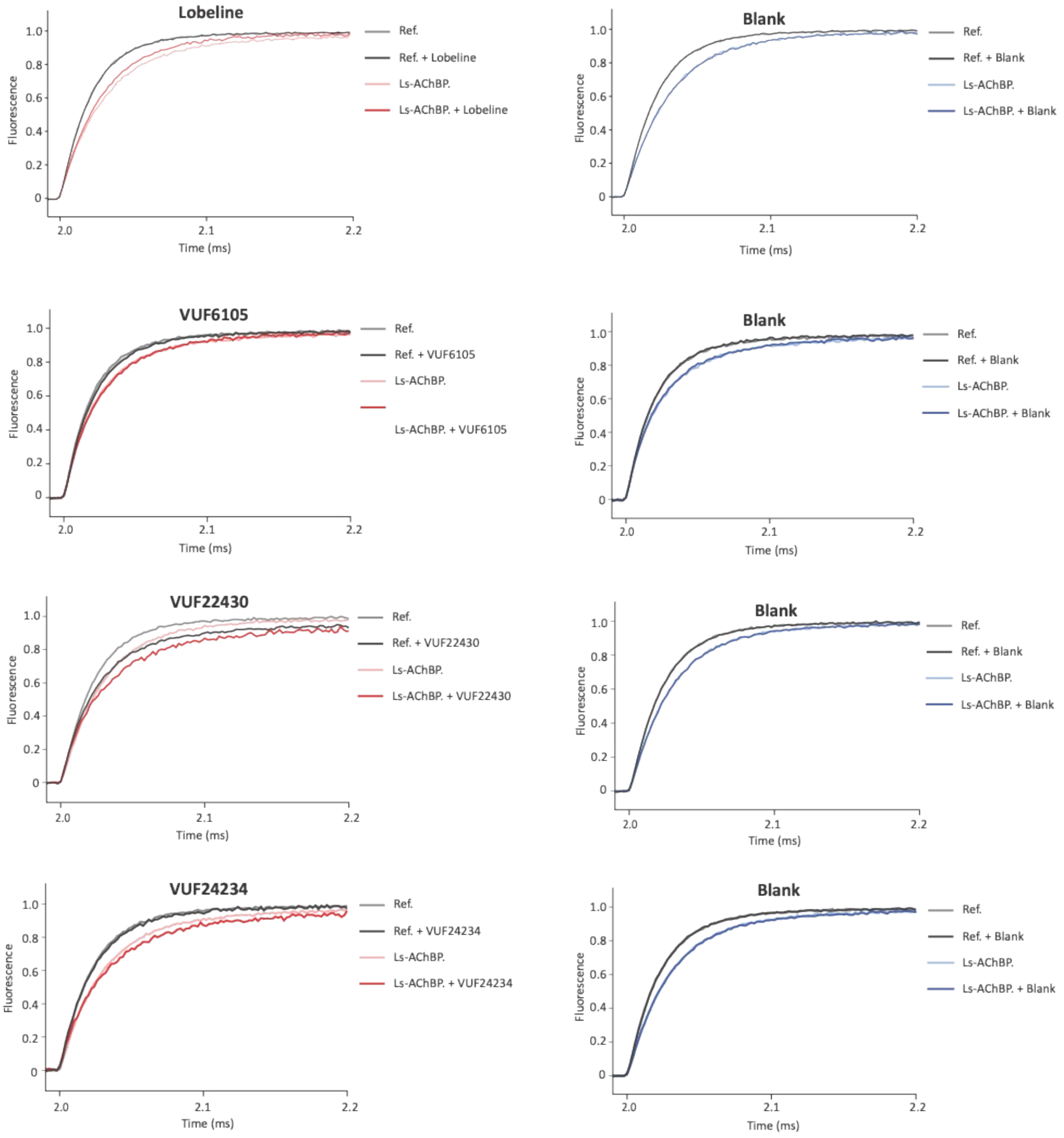
Lobeline
Ref.
Ref. + Lobeline
Ls-AChBP.
Ls-AChBP. + Lobeline
Blank
Ref.
Ref. + Blank
Ls-AChBP.
Ls-AChBP. + Blank
VUF6105
Ref.
Ref. + VUF6105
Ls-AChBP.
Ls-AChBP. + VUF6105
VUF22430
Ref.
Ref. + VUF22430
Ls-AChBP.
Ls-AChBP. + VUF22430
VUF24234
Ref.
Ref. + VUF24234
Ls-AChBP.
Ls-AChBP. + VUF24234
Fluorescence
Time (ms)
0
0.2
0.4
0.6
0.8
1.0
2.0
2.1
2.2

Figure S6. Crystal structures of AChBP in complex with ligands

Top row: VUF24234 in the monomer interface, shown as (a) top view, (b) side view, and (c) close up view. Middle and bottom rows: (d) VUF6105, (e) FL1888, (f) FL1856, (g) FL1613, (h) FL3044 and (i) FL1909 in the monomer interface.

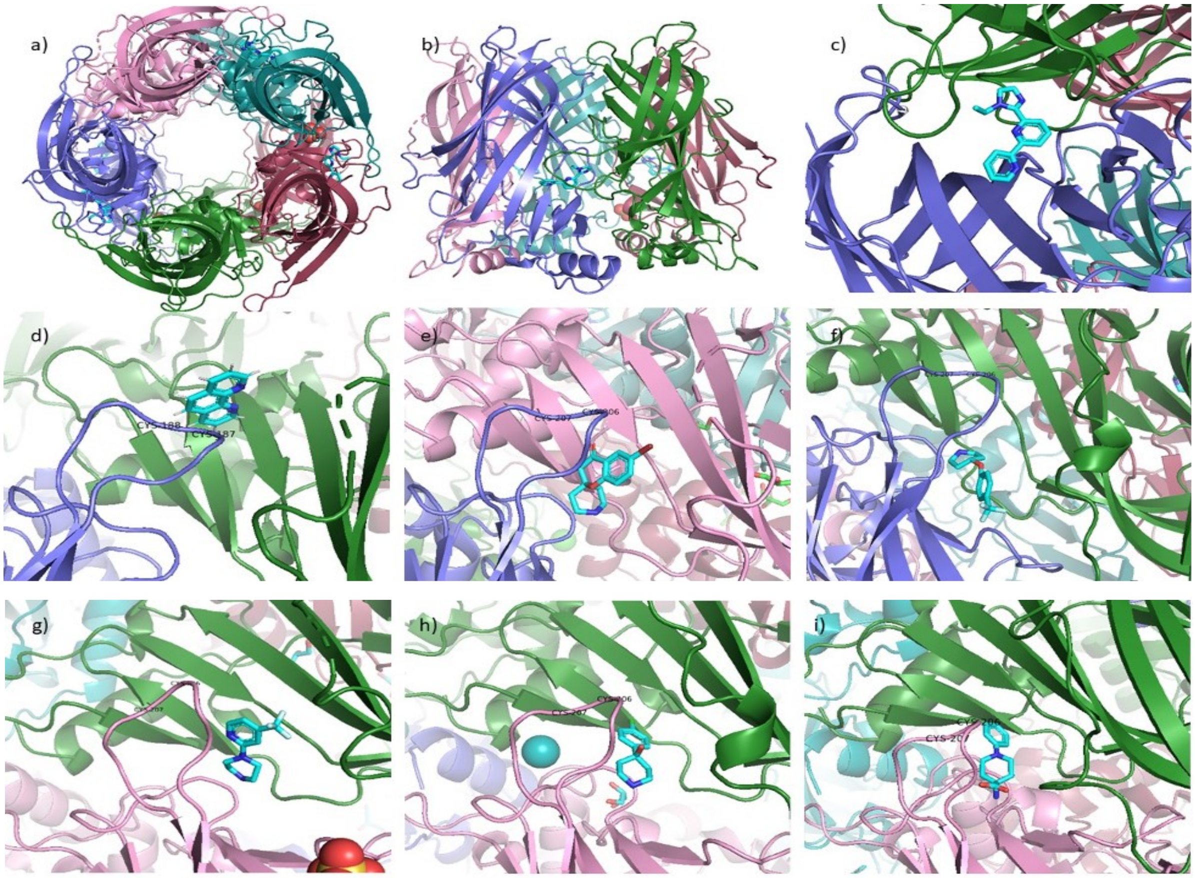

Table S1. Data collection and refinement statistics for crystal structures of AChBP and FL-compounds

**Compound**	**FL1909**	**FL3044**	**FL1613**
PDB ID	8P1F	8P11	8P1E
**Data collection**	DLS, I04	DLS, I04	MAX IV, BioMAX
Detector	DECTRIS EIGER2 16M	DECTRIS EIGER2 16M	DECTRIS EIGER2 16M
Space group	$P1\ 2_1\ 1$	$P\ 2_1\ 2_1\ 2_1$	$P\ 2_1\ 2_1\ 2_1$
Wavelength (Å)	0.979	0.979	0.976
Unit cell a, b, c (Å)	116.71, 140.71, 147.17	76.25, 120.64, 240.01	77.10, 121.08, 241.21
α, β, γ (°)	90.00, 109.68, 90.00	90.00, 90.00, 90.00	90.00, 90.00, 90.00
Resolution range (Å)	49.71 - 2.1 (2.175 - 2.1)	48.16 - 1.9 (1.968 - 1.9)	48.36 - 2.1 (2.175 - 2.1)
Total reflections (#)	1837977 (181413)	2414545 (244048)	1791330 (182433)
Unique reflections (#)	259407 (25710)	174607 (17257)	132342 (13095)
Multiplicity	7.1 (7.1)	13.8 (14.1)	13.5 (13.9)
Completeness (%)	99.68 (99.26)	99.96 (99.98)	99.96 (99.96)
R_{merge}	0.0742 (1.471)	0.1094 (2.451)	0.3436 (3.099)
R_{meas}	0.08002 (1.587)	0.1136 (2.542)	0.3577 (3.216)
R_{pim}	0.02981 (0.5931)	0.03031 (0.6693)	0.09844 (0.8544)
Mean $I/\sigma(I)$	15.68 (1.28)	16.40 (0.98)	9.73 (1.02)
$CC_{1/2}$	0.999 (0.511)	1 (0.491)	0.99 (0.537)
Wilson *B*-factor (Å^2)	50.23	37.74	42.71
**Refinement**			
Reflections used in refinement	259344 (25709)	174559 (17257)	132318 (13095)
Reflections used for R_{free}	12969 (1286)	8729 (863)	6617 (655)
R_{work}	0.2239 (0.3499)	0.2233 (0.3917)	0.2119 (0.3419)
R_{free}	0.2490 (0.3630)	0.2429 (0.3767)	0.2397 (0.3617)
# non-hydrogen atoms	33433	16962	16973
Macromolecules	32499	16296	16268
Ligands	230	16	125
Solvent	704	650	580
Protein residues	4039	2024	2023
r.m.s.d			
bond lengths (Å)	0.014	0.014	0.014
bond angles (˚)	1.90	1.83	1.86
Ramachandran plot, residues in			
favoured (%)	98.76	99.40	99.45
allowed (%)	1.06	0.60	0.50
outliers (%)	0.18	0.00	0.05
Rotamer outliers (%)	1.26	0.79	0.16
Clashscore	3.96	3.49	2.05
Average *B*-factor	62.36	47.85	51.45
Macromolecules (Å^2)	62.28	48.01	51.42
Ligands (Å^2)	110.14	42.78	76.05
Solvent (Å^2)	50.05	44.06	46.94

Statistics for the highest-resolution shell are shown in parentheses.

Table S2. Data collection and refinement statistics for crystal structures of AChBP and VUF-compounds

**Compound**	**VUF24234**	**VUF6105**
PDB ID	8P22	9SG3
**Data collection**	MAX IV, BioMAX	DLX, I04
Detector	DECTRIS EIGER2 16M	DECTRIS EIGER2 16M
Space group	*C 1 2 1*	$P2_12_12_1$
Wavelength (Å)	0.9762	0.979
Unit cell a, b, c (Å)	143.96, 120.6, 133.16	74.95, 117.85, 239.75
α, β, γ (°)	90, 91.499, 90	90.00, 90.00, 90.00
Resolution range (Å)	49.51 - 2.2 (2.279 - 2.2)	49.59 - 3.0 (3.107 - 3.0)
Total reflections (#)	202225 (20560)	325476 (31167)
Unique reflections (#)	101963 (10549)	43398 (4270)
Multiplicity	2.0 (1.9)	7.5 (7.3)
Completeness (%)	88.32 (91.57)	99.89 (99.93)
R_{merge}	0.1379 (0.6544)	0.2174 (1.906)
R_{meas}	0.1852 (0.8643)	0.2334 (2.052)
R_{pim}	0.1227 (0.5597)	0.08419 (0.7528)
Mean $I/\sigma(I)$	7.41 (1.12)	8.28 (1.05)
$CC_{1/2}$	0.907 (0.588)	0.997 (0.523)
Wilson *B*-factor (Å^2)	45.63	78.11
**Refinement**		
Reflections used in refinement	101774 (10549)	43375 (4269)
Reflections used for Rfree	5089 (528)	1999 (197)
R_{work}	0.2381 (0.4053)	0.2476 (0.4254)
R_{free}	0.3038 (0.4053)	0.2691 (0.4364)
# non-hydrogen atoms	16816	16393
Macromolecules	16008	16110
Ligands	173	108
Solvent	635	175
Protein residues	1998	2014
r.m.s.d.		
bond lengths (Å)	0.014	0.004
bond angles (˚)	1.93	0.85
Ramachandran plot, residues in		
favored region (%)	95.97	95.64
allowed region (%)	3.73	3.75
outlier region (%)	0.31	0.61
Rotamer outliers (%)	4.46	0.48
Clashscore	7.82	9.19
Average *B*-factor	50.55	79.79
Macromolecules (Å^2)	50.66	79.59
Ligands (Å^2)	54.17	129.71
Solvent (Å^2)	46.67	67.22

Statistics for the highest-resolution shell are shown in parentheses.
